# Improving the treatment of bacterial infections caused by multidrug-resistant bacteria through drug repositioning

**DOI:** 10.3389/fphar.2024.1397602

**Published:** 2024-06-07

**Authors:** Paulina Glajzner, Agnieszka Bernat, Magdalena Jasińska-Stroschein

**Affiliations:** Department of Biopharmacy, Faculty of Pharmacy, Medical University of Lodz, Łódź, Poland

**Keywords:** drug repositioning, bacterial infections, antimicrobial drug resistance, carbapenem-resistant enterobacteriaceae, methicillin resistance, tuberculosis

## Abstract

Drug repurposing (repositioning) is a dynamically-developing area in the search for effective therapy of infectious diseases. Repositioning existing drugs with a well-known pharmacological and toxicological profile is an attractive method for quickly discovering new therapeutic indications. The off-label use of drugs for infectious diseases requires much less capital and time, and can hasten progress in the development of new antimicrobial drugs, including antibiotics. The use of drug repositioning in searching for new therapeutic options has brought promising results for many viral infectious diseases, such as Ebola, ZIKA, Dengue, and HCV. This review describes the most favorable results for repositioned drugs for the treatment of bacterial infections. It comprises publications from various databases including PubMed and Web of Science published from 2015 to 2023. The following search keywords/strings were used: drug repositioning and/or repurposing and/or antibacterial activity and/or infectious diseases. Treatment options for infections caused by multidrug-resistant bacteria were taken into account, including methicillin-resistant staphylococci, multidrug-resistant *Mycobacterium tuberculosis*, or carbapenem-resistant bacteria from the *Enterobacteriaceae* family. It analyses the safety profiles of the included drugs and their synergistic combinations with antibiotics and discusses the potential of antibacterial drugs with antiparasitic, anticancer, antipsychotic effects, and those used in metabolic diseases. Drug repositioning may be an effective response to public health threats related to the spread of multidrug-resistant bacterial strains and the growing antibiotic resistance of microorganisms.

## 1 Introduction

For several years, an increase in antibiotic resistance of bacterial strains has been observed in various types of infections, both hospital and community-acquired, and in various age groups (Wattal and Goel, 2020). One of the most important problems in the treatment of infections is the occurrence of multidrug-resistant bacteria, where resistance affects one (multidrug-resistance, MDR), several (extensively drug-resistant, XDR) or all (pandrug-resistance, PDR) groups of the used antibacterial drugs (Magiorakos et al., 2012). The highest priority pathogens include both Gram-negative and Gram-positive bacteria (Fritsche, 2021). They constitute the ESKAPE group of alert pathogens (E—*Enterococcus faecium*, S—*Staphylococcus aureus*, K—*Klebsiella pneumoniae*, A—*Acinetobacter baumannii*, P—*Pseudomonas aeruginosa*, E—*Enterobacter* spp.), which constitute the greatest threat in the case of nosocomial infections (Loyola-Cruz et al., 2023). The key pathogens, in addition to *K. pneumoniae*, also include other genera from the *Enterobacteriaceae* family—*Escherichia coli*, *Serratia* sp., and *Proteus* sp. (WHO, 2017; Asokan et al., 2019). In recent years, the problem of drug resistance has also affected bacteria of the *Mycobacterium* genus. Tuberculosis has also become a major global health problem. Cases of extensively drug-resistant tuberculosis (XDR-TB) have been reported in over 100 countries around the world (Kanvatirth et al., 2019; Kaya et al., 2023).

The problem of antibiotic resistance results, among others, from their unjustified application, use in inappropriate clinical situations, and in inappropriate doses for the treatment of infections. In addition to natural antibiotic resistance, as a result of the improper use of antibiotics, bacteria have developed various mechanisms as a result of mutations in chromosomal genes or genes constituting mobile genetic elements and as a result of horizontal gene transfer. This is related to the phenomenon of selective pressure among microorganisms (Bassetti et al., 2022; Hryniewicz and Struzycka, 2023). However, the development of bacterial resistance to antibiotics due to genome changes may occur not only due to inappropriate drug applications but also independently of them. Multidrug-resistant bacteria are estimated to cause 25,000 deaths per year in Europe (Morehead and Scarbrough, 2018).

In recent years, the number of new antibiotics approved for use in medicine has been insufficient, given the growing problem of drug resistance. A 2021 WHO analysis found that 217 antibacterial drugs were in preclinical development. However, according to the WHO report from 2022, in 2017–2021 only 12 new antibiotics were registered and approved for use (WHO, 2022). The answer to the problem of too few new, effective antibiotics may be drug repositioning. This process is based on the use of old drugs belonging to different therapeutic classes in a new medical indication (Foletto et al., 2021a). It provides a solution to the high costs and slow process of discovering new drugs (Hua et al., 2022). Repositioning allows bypassing many stages on the way to the registration of a new drug due to the known pharmacokinetic and toxicity profiles of the drug. In the case of repositioned drugs, the preclinical phase is only related to demonstrating their effectiveness in a new indication in a cellular or animal model (Farha and Brown, 2019; Kanvatirth et al., 2019).

Various techniques are used to discover a new application for a drug. One of them includes molecular modeling techniques (including, among others, molecular docking, molecular dynamic simulations and quantitative structure activity relationships), which allows predicting biological activity and virtual screening of molecules (Kumar et al., 2022). Important tools in the process of drug repositioning are databases that contain pathways connecting genes and proteins responsible for biological processes that may be influenced by the interactions of drugs with their therapeutic targets (Pan et al., 2014; Gonzalez-Cavazos et al., 2023).

Drugs that have been successfully repositioned include: bupropion, originally used to treat depression and now also used in the treatment of addictions (tobacco smoking); sildenafil, which was originally registered for treatment of pulmonary arterial hypertension and is now also indicated for erectile dysfunction; or minoxidil registered as a drug for hypertension, currently used to treat androgenic alopecia (Ashburn and Thor, 2004; Farha and Brown, 2019). Currently, research on the repositioning of drugs for the treatment of viral diseases is developing rapidly. In recent years, this has also been demonstrated by the pandemic caused by SARS-CoV-2 (Hua et al., 2022; Vaz et al., 2023). Research into drugs repositioned for the treatment of ZIKA virus infections include substances such as mycophenolic acid used as an immunomodulator and memantine used to treat Alzheimer’s disease. Repositioning clomiphene and toremifene, used in the treatment of breast cancer and infertility, for the treatment of Ebola virus infection is also being considered (Sirohi and Kuhn, 2017; Mani et al., 2019).

Due to the spread of multidrug-resistant strains, the possibility of changing the purpose of classes of non-antibiotic drugs in the treatment of severe bacterial infections is also emphasized (Foletto et al., 2021a; Caldara and Marmiroli, 2021). This review focuses on the potential repurposing of several drugs and their use in the treatment of bacterial infections. Articles published between 2015 and 2023 were searched, including *in vitro* and *in vivo* studies of drug candidates for repositioning. Keywords included: drug repositioning and/or repurposing and/or antimicrobial activity and/or infectious diseases. The applied search strategy allowed for the selection of nearly 1,700 publications. The focus was on work taking into account the spectrum of activity of the tested potential drug candidates in the case of bacterial infections. Works on viral and fungal infections were not included. Drug structures, probable mechanisms of action, and bacterial species used in the research were taken into account. Attention was also paid to the role of synergism in the case of combining tested drugs and antimicrobial substances, which would allow for dose reduction and would reduce the selection pressure among bacteria. These applied criteria allowed the search for 113 scientific articles that were used to write this manuscript. The Clinical Trials database was also searched (studies reported since 2015), where the keywords were the names of active substances included in the manuscript.

## 2 Potential candidates for repositioning in the treatment of bacterial infections

**FIGURE 1:**
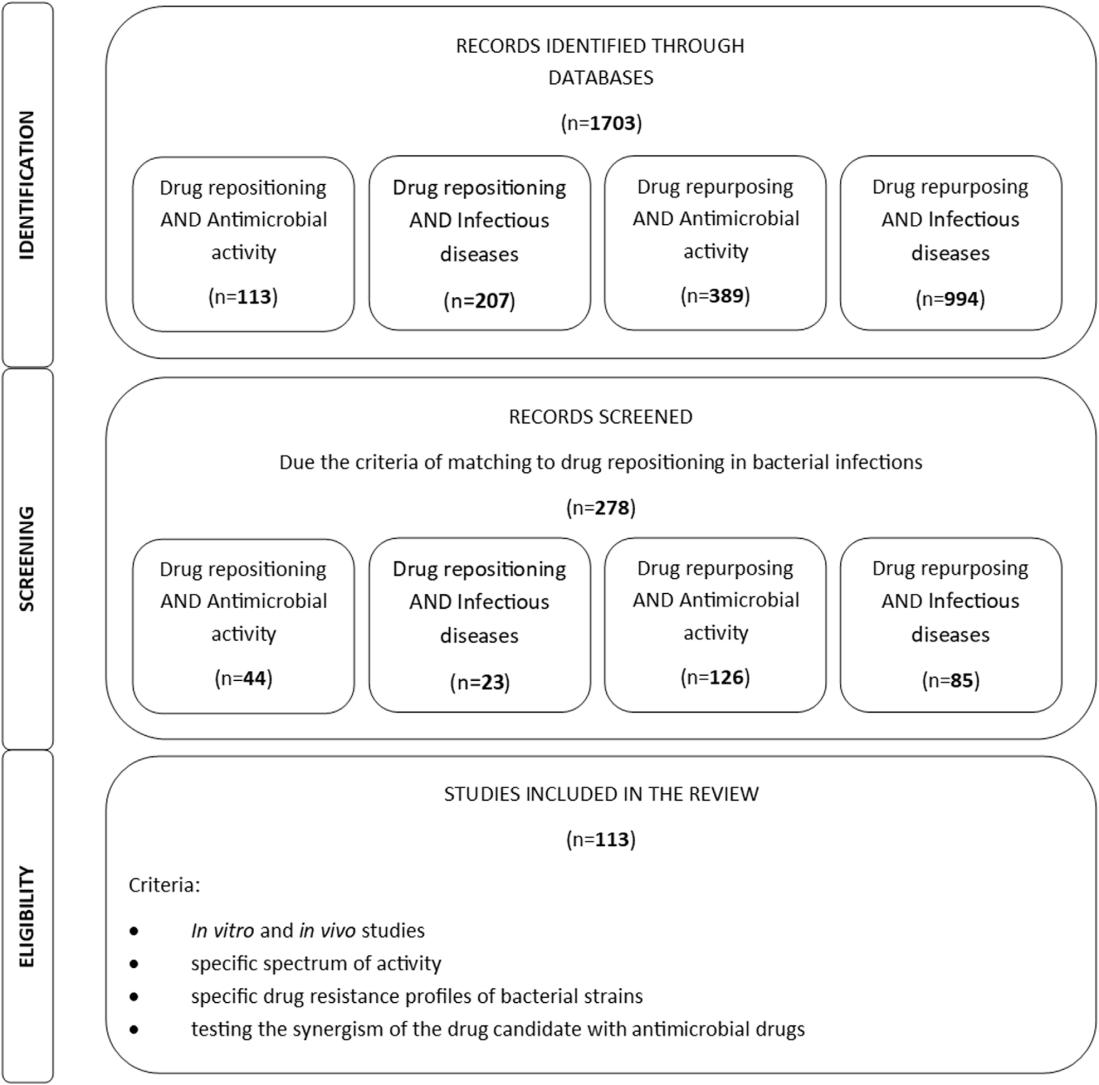
Results of the applied article search strategy.

Research on drug repositioning in antimicrobial therapy is currently developing rapidly. The applied article search strategy allowed us to filter over 1,700 scientific articles. Figure 1 presents the strategy and summary of the literature search results. The multitude of articles published in recent years indicates a growing interest in the topic of drug repositioning in infectious diseases. According to many of them, which concern repositioning not only in bacterial diseases, but also research is ongoing in fungal and viral diseases, which is also related to the recent COVID-19 pandemic.

More than 70 out of 1,600 non-antimicrobial drugs with antimicrobial activity were selected in *in vitro* tests in one of the screening studies aimed at discovering new antibacterial drugs. They were active against at least one pathogen from the ESKAPE group (Younis et al., 2017). Anti-inflammatory drugs, antidepressants, antihypertensives, statins, proton pump inhibitors have been recognized as potential candidates in the process of drug repositioning in the treatment of infectious diseases (da Rosa et al., 2022).

### 2.1 Medicines used in oncology

#### 2.1.1 Cytostatics

Candidates for antibacterial drugs include cytotoxic and anticancer drugs. However, due to their cytotoxicity or genotoxicity profile, they can be considered as having high clinical value, mainly in the treatment of infections in oncological patients (Gouveia et al., 2023).

One of the concerned anticancer substances is floxuridine, a derivative of 5-fluorouracil, which has demonstrated antibacterial activity against *S. aureus* strains, including methicillin resistance *S. aureus* (MRSA), and *S. epidermidis*. It is important in research on this drug to optimize the structure of the drug molecule so that it is not activated in human cells and does not have a cytotoxic effect on them (Thangamani et al., 2015; Younis et al., 2017; Cheng et al., 2019; Hua et al., 2022). Ethyl bromopyruvate, a derivative of 3-bromopyruvic acid, demonstrated antibacterial activity against strains of the ESKAPE group. The authors of the study proved that 24-h bromopyruvate therapy can achieve the same effects as vancomycin therapy. Additionally, this compound was responsible for inhibition of iron uptake, by *Mycobacterium tuberculosis* (Kumar et al., 2019a).

It has also been proven that oncological drugs can be effective in combination therapy with antibiotics. Mitoxantrone, in combination with colistin was effective in the treatment of *P. aeruginosa* infections and biofilm control (Torres et al., 2018). In turn, mitotane with polymyxin B resulted in an increased antibacterial activity against isolates resistant to this antibiotic and carbapenem-resistant *A. baumannii*, *P. aeruginosa*, and *K. pneumoniae*. In this combination, polymyxin B, due to its action on the outer membrane of Gram-negative bacterial cells, allowed the oncological drug to penetrate into them (Tran et al., 2018).

Research on the repositioning of this group of drugs in infectious diseases is currently under investigation in the preclinical phase. At present, clinical trials on the repositioning of anticancer drugs mainly concern the treatment of Alzheimer’s disease. They are believed to have the ability to regulate processing and reduce the aggregation of amyloid plaques (Advani and Kumar, 2021). It has been shown that the use of these drugs in oncological diseases reduced the risk of developing dementia (Ancidoni et al., 2021).

#### 2.1.2 Tyrosine kinase inhibitors

The development of tyrosine kinase inhibitors (TKIs) represents one of the most significant milestones in the medicine of the 21st century. The first TKI, imatinib, was designed to target the BCR-Abl hybrid protein, produced in patients with Philadelphia-chromosome-positive chronic myelogenous leukemia. Since the introduction of imatinib, the application of TKIs has been ever-expanding, but has addressed mainly oncological patients (Thomson et al., 2023). Several signaling pathways have gained attention as potential targets for repurposing of TKIs for other diseases; these include pulmonary arterial hypertension (PAH), as a platelet-derived growth factor receptor, or mast/stem cell growth factor receptor kit (c-KIT), can contribute to inflammation and proliferation processes being involved in the pathogenesis of the disease. The results from experimental studies with imatinib were promising, and oral application yielded improvements in exercise capacity and hemodynamics in clinical trials; however its further development was limited by systemic side effects with subdural hematomas (Weatherald et al., 2020). Currently, research is underway on the repositioning of TKIs in the treatment of Human Immunodeficiency Virus (HIV) infection. *In vitro* studies have shown that TKIs have the ability to modulate the immune response and have antiviral activity against not only HIV-1 virus but also Cytomegalovirus (CMV), Monkeypox virus (MPV), Varicella zoster virus (VZV), and filoviruses. The greatest hopes are associated with the repositioning of TKIs in viral diseases due to the inhibition of the escape of viruses from infected cells, thus reducing the spread of infection (Ananthula et al., 2018; Climent and Plana, 2019; Vigón et al., 2020; Rodríguez-Agustín et al., 2023).

Research on the antibacterial activity of TKIs is currently in the preclinical phase; however, studies have identified two candidates. Fostamatinib was considered a molecule with anti-tuberculosis activity. It inhibits the activity of protein kinases (serine/threonine-protein kinase) found in *M. tuberculosis* and *M. bovis*. This drug was found to kill infected macrophages and did not show cytotoxicity (Rodrigues et al., 2020). Ponatinib also demonstrated antibacterial activity against planktonic cells and biofilm-forming *Streptococcus mutans* (Saputo et al., 2018).

#### 2.1.3 Hormonal drugs—selective estrogen receptor modulators

Selective estrogen receptor modulators (SERMs) are used in the treatment of cancer and in the prevention of osteoporosis in postmenopausal women. Examples of SERMs are tamoxifen, toremifene, raloxifene, and clomiphene. In addition to its listed indications, tamoxifen is also used to treat infertility and reduce the risk of breast cancer in women. These drugs compete with estrogen and prevent its binding to the estrogen receptor, thus inhibiting its stimulating effect. They also induce apoptosis and regulate the expression of oncogenes and growth factors. Moreover, they also demonstrate various pleiotropic neuroprotective, cardioprotective, and antimicrobial effects (Garg et al., 2020).

The SERM clomiphene demonstrated antibacterial activity against strains of *Bacillus subtilis*, *E. faecium* and methicillin-resistant staphylococci. It is believed to act by influencing a bacterial cytoplasmic enzyme important for the synthesis of teichoic acid. Its inhibition leads to impaired cell wall synthesis of Gram-positive bacteria. In the case of Gram-negative bacteria, clomiphene have been found to have an effect in combination with colistin. In these bacteria, this SERM member appears to influence the synthesis and transport of lipopolysaccharide precursors (Younis et al., 2017; Torres et al., 2018).

Toremifene has also demonstrated antibacterial activity. When applied in combination with polymyxin B, it is characterized by a strong antibacterial effect against *P. aeruginosa* bacterial cells, which form biofilms and are usually resistant to these antibiotics. Toremifene induced depolarization of the cytoplasmic membrane, increased permeabilizing activity and stimulated the production of reactive oxygen species in bacterial cells. It is believed that by influencing the permeability of bacterial cell membranes and lipopolysaccharides, polymyxins enable SERMs to better penetrate microbial cells. Unlike the previously-mentioned SERMs, tamoxifen interacts with lipids in bacterial membranes, causing structural changes that may result in cell lysis. Its analogues, however, induce the outflow of ions from Gram-negative bacterial cells and lead to the loss of their membrane potential (Hussein et al., 2017). In addition, SERM drugs have also shown activity against viruses, fungi, and parasites (Johansen et al., 2013; Garg et al., 2020). One clinical study assessed the safety and effectiveness of tamoxifen in combination with amphotericin B and fluconazole in the treatment of cryptococcal meningitis (NCT03112031), but failed to obtain satisfactory results.

**FIGURE 2:**
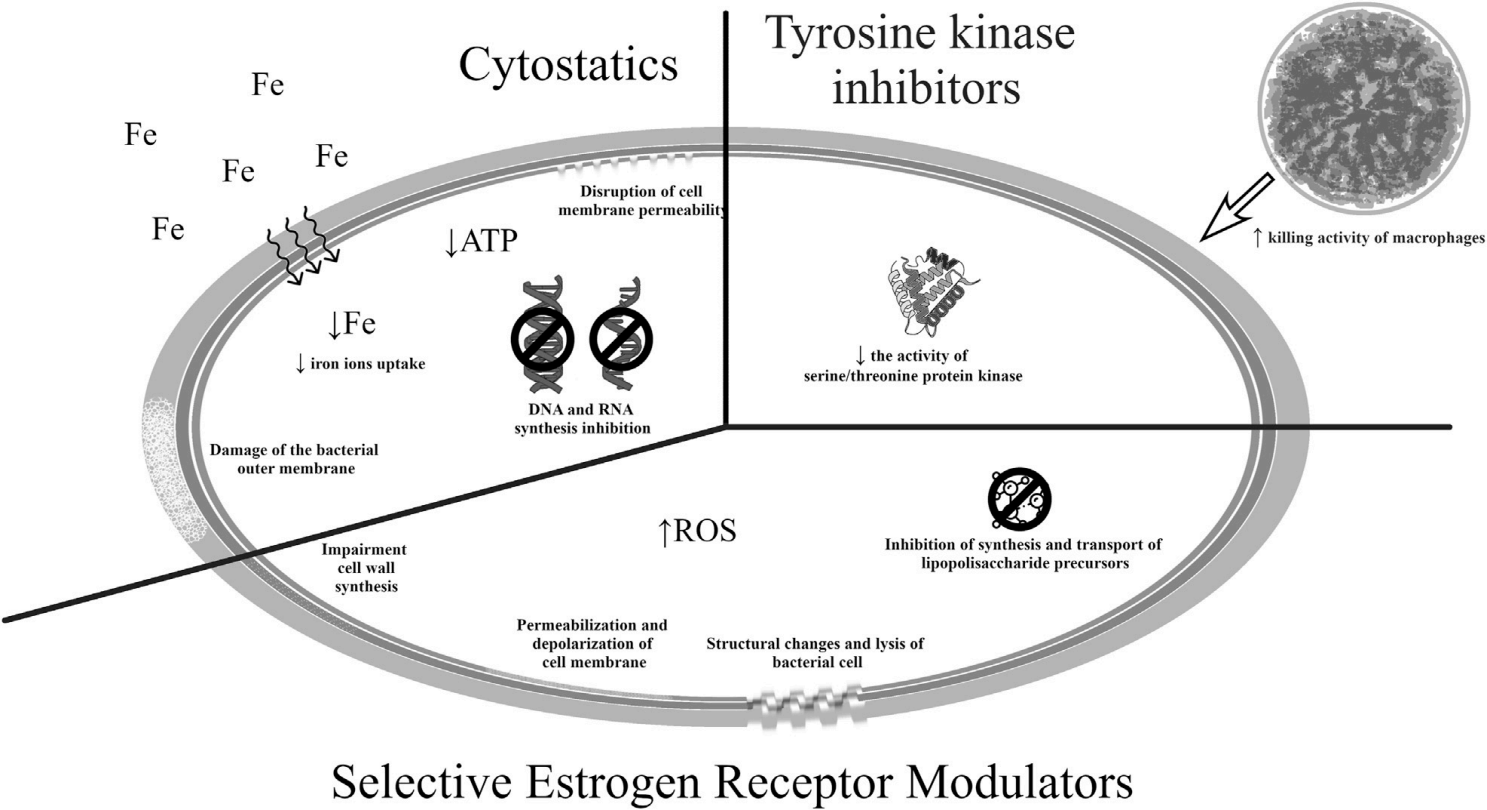
Potential mechanisms of antibacterial action of medicines used in oncology.

Potential mechanisms of action of the drugs listed in this section are presented in Figure 2 (Johansen et al., 2013; Younis et al., 2017; Saputo et al., 2018; Torres et al., 2018; Tran et al., 2018; Garg et al., 2020; Advani and Kumar, 2021; Li et al., 2023).

### 2.2 Medicines used in gastroenterology

Proton pump inhibitors (PPI), typically used in the treatment of gastric hyperacidity, stomach ulcers, gastroesophageal reflux and gastritis, also inhibit the growth of bacteria, fungi and viruses. They have demonstrated antibacterial activity against Gram-positive bacteria (*S. aureus*, *E. faecalis*) and a number of Gram-negative bacteria (*P. aeruginosa*, *E. coli*, *A. baumannii*, *K. pneumoniae*, *E. cloacae*, *U. urealyticum*), as well as *M. tuberculosis*. Molecules with antimicrobial activity include esomeprazole, lansoprazole, omperazole, and pantoprazole (Foletto et al., 2021a; da Rosa et al., 2022). It has been proven that PPIs inhibit the activity of fluoroquinolone efflux pumps in *S. aureus* strains, which increases the activity of, among others, levofloxacin, ciprofloxacin and norfloxacin in staphylococcal infections (Thangamani et al., 2015). Additionally, when used in therapy, they may also interrupt the respiratory chain and deplete energy resources, as demonstrated in studies on mycobacteria (Rodrigues et al., 2020).

Currently, the possibility of repositioning proton pump inhibitors is under examination in many preclinical and clinical studies. One clinical trial involved the use of esomeprazole to reduce organ failure in sepsis (NCT03452865), but no results have been posted so far. This molecule has been shown to inhibit the secretion of TNF-α and IL-1β. In studies in animal models, a single dose of esomeprazole protected against endotoxic shock. Other studies include the possibility of using PPIs in cancer treatment due to their ability to inhibit fatty acid synthase, induce cancer cell apoptosis, and inhibit the proton pump of cancer cells (Ikemura et al., 2017; Spugnini and Fais, 2020). Clinical trials are currently in the recruitment phase (NCT04930991, NCT04337580).

The opioid loperamide, a drug that inhibits gastrointestinal motility is poorly absorbed and well tolerated. It is used in the treatment of infectious and non-infectious chronic and acute diarrhea (Brown, 2015; Zhang et al., 2023). Due to its mechanism of action, i.e., mediating the intracellular calcium concentration by blocking calcium channels and activating opioid receptors, it is also a candidate for relieving pain, controlling anxiety or reducing insulin resistance (Juárez et al., 2018; Barrientos et al., 2021). As an agent with antibacterial potential, loperamide has demonstrated synergism with several classes of antibiotics in the treatment of bacterial infections causing intestinal infections (*in vitro* studies). Its use increased the effectiveness of tetracyclines, cephalosporins, as well as polymyxin B and colistin against Gram-negative bacteria. The molecule has been recognized to influence the deformation of bacterial cells and the permeability of their cell membrane, which may allow the antibiotic to accumulate. For this reason and due to the pharmacokinetic properties of this drug, some authors claim that loperamide can be used in the treatment of intestinal infections (Brown, 2015; Zhang et al., 2023). Moreover, *in vitro* tests have demonstrated bactericidal activity against the tuberculous strain *M. tuberculosis* and the non-tuberculous *M. bovis* BCG, *M. terrae* and *M. smegmatis*. In addition to activating bactericidal mechanisms, this drug also caused an immune response, influencing the activity of macrophages and the inflammatory process (Juárez et al., 2018; Barrientos et al., 2021). Research on the antibacterial effect of loperamide is currently in the preclinical phase.

**FIGURE 3:**
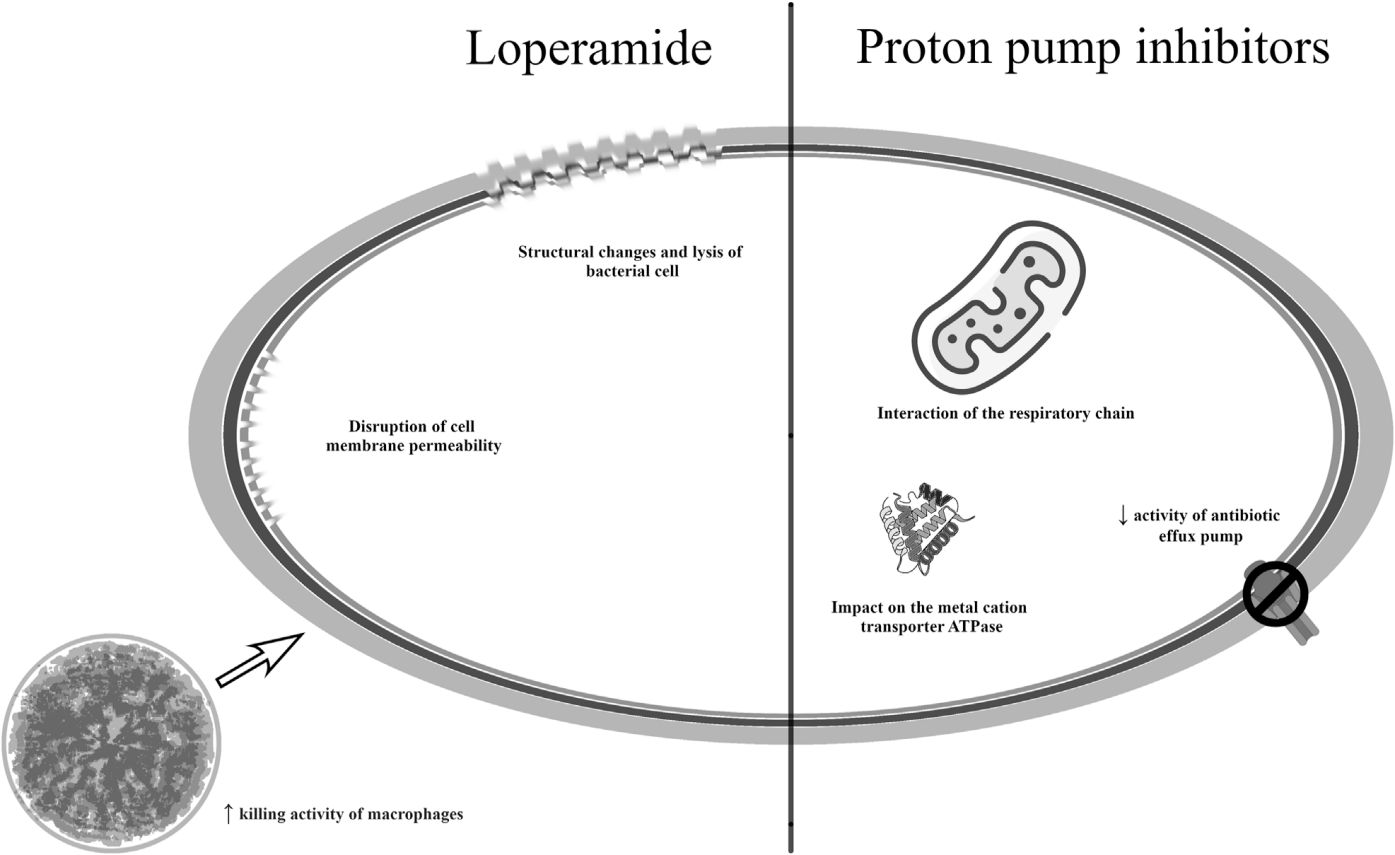
Potential mechanisms of antibacterial action of medicines used in gastroenterology.

Figure 3 shows the potential mechanisms of action of the drugs mentioned in this section (Thangamani et al., 2015; Juárez et al., 2018; Saputo et al., 2018; Barrientos et al., 2021; Zhang et al., 2023).

It can be assumed that drugs used in gastroenterology may potentially be repositioned as supportive therapy for antibiotic treatment in infectious diseases of the gastrointestinal tract and sepsis.

### 2.3 Medicines used in therapy against pain

#### 2.3.1 Non-steroidal anti-inflammatory drugs

Non-steroidal anti-inflammatory drugs (NSAIDs) are one of the most frequently-prescribed therapeutic groups, with the “basic” mode of action covering the inhibition of COX enzymes and reduction of release of prostaglandins (PGs) and thromboxane (TxA). While inhibition of COX isoforms has been demonstrated as a primary mechanism for the efficacy of the NSAIDs, preclinical and clinical data have revealed the presence of additional COX-independent mechanisms. These include the inhibition of various transcription factors: activator protein 1 (AP-1) and nuclear factor-kappa B (NF-ĸB) regulating the expression of various pro-inflammatory cytokines (TNFα, IL-1β, IL6) and mediators (NO, PGE2, ICAM-1, VCAM-1) inhibiting signaling pathways involved in inflammatory processes (MAPK and PI3K/AKT) modulating nuclear receptors (PPAR-γ and PPAR-δ) inhibiting leukocyte and neutrophil adherence, inhibiting matrix metalloproteinases (MMP2 and MMP9) or inhibiting cAMP-specific phosphodiesterase (PDE) IV. Some of these mechanisms may support the rationale for *in vitro*/*in vivo* studies on the antiproliferative, antimetastatic, antiangiogenesis, and neuroprotective effects of NSAIDs. Due to these multifactorial effects, they may represent promising candidates for repurposing for chemoprevention and cancer treatment (Ozleyen et al., 2023).

Antibacterial activity has been demonstrated, among others, by acetylsalicylic acid, diclofenac, ibuprofen, celecoxib and fenamic acids. While indomethacin, meloxicam, naproxen, nimesulid, and paracetamol have also demonstrated antibacterial activity, the possibility of using them in the treatment of infections requires further research (Foletto et al., 2021a). Acetylsalicylic acid (ASA) inhibited the production of alpha toxin and negatively influenced the expression of genes responsible for adhesion to components of the host extracellular matrix in *S. aureus* strains, including methicillin-resistant ones (Barbarossa et al., 2022). ASA has been found to have a synergistic effect with vancomycin in an animal model of endocarditis (Brown, 2015). Currently, studies are underway to use the indicated anti-inflammatory drug to reduce mortality in HIV-infected tuberculosis patients (NCT04145258, phase 3 clinical trial, currently recruiting) (Maitre et al., 2022). Another example of an NSAID drug that has demonstrated antimicrobial activity is ibuprofen. It inhibited the growth of *E. coli* and *P. aeruginosa*, as well as *S. aureus* strains under low pH conditions. Additionally, ibuprofen also reduced the rate of bacterial cell accumulation during biofilm formation by *P. aeruginosa* cells (Barbarossa et al., 2022). An ongoing clinical trial, currently in the recruitment phase, is focusing on the use of acetylsalicylic acid and ibuprofen as adjunctive therapy in the treatment of both sensitive and multidrug-resistant tuberculosis (NCT04575519).

Diclofenac is another molecule of interest for studies on drug repurposing due to its properties to inhibit biofilm formation on the dentin after topical usage (Barbarossa et al., 2022). Studies have also shown that it might inhibit the number of viable *E. coli* and *Mycobacterium* cells when given in combination with streptomycin, and to have an antibacterial effect against *Listeria* spp. strains when given with gentamycin (Brown, 2015; Foletto et al., 2021a). *In vitro* studies also found combined diclofenac and oxacillin to be effective against MRSA strains. The use of these two substances led to apoptosis of bacterial cells through damage to the cell membrane and breaks in the DNA (Queiroz et al., 2021). Diclofenac also inhibited the multiplication of MDR clinical isolates of *E. coli*, *A. baumannii*, *S. aureus*, and *S. epidermidis* (Foletto et al., 2021a); however, its antibacterial effects were only observed at very high doses (Brown, 2015). Research is currently underway on its new derivatives with better properties (Hamed et al., 2023; Tolba et al., 2023). So far, the use of diclofenac has also been considered in the treatment of various types of cancer (Pantziarka et al., 2016).

Celecoxib has demonstrated antibacterial activity against both Gram-negative and Gram-positive bacterial strains. It improved the sensitivity of *M. smegmatis* and MRSA strains to antimicrobial therapy by increasing the uptake of antibiotics by bacterial cells; this was observed when the drug was used either locally or systemically. Depending on the dose used, celecoxib could also inhibit RNA, DNA and proteins synthesis and reduce the level of pro-inflammatory cytokines (Bak and Krupa, 2023). Interestingly, it has also been shown to be effective against *Francisella tularensis* strains causing tularemia (Chiu et al., 2009). Research conducted to determine effective and safe doses of celecoxib in antibacterial therapy identified structural analogues with strong antimicrobial activity. These molecules were able to improve the effectiveness of polymyxins against multidrug-resistant Gram-negative bacteria and were active against MRSA strains (Krishnamurthy et al., 2019). Current clinical trials are focused on evaluating the activity of celecoxib in the treatment of tuberculosis (NCT02602509, completed, no study results) and in viral suppression in chronic hepatitis B (NCT05256823, pre-recruitment). In addition to its antimicrobial activity, celecoxib has also shown good results as a possible drug in anticancer therapy (Bak and Krupa, 2023).

Fenamic acid derivatives, tolfenamic acid, flufenamic acid, and mefenamic were effective against *Neisseria gonorrhoeae* strains. Importantly, these compounds did not affect commensal *Lactobacillus* spp. Fenamic acid derivatives showed better properties in reducing the intracellular load of *N. gonorrhoeae* in infected cells compared to ceftriaxone and reduced the expression of pro-inflammatory cytokines in cervical cells (Seong et al., 2020).

The use of nonsteroidal anti-inflammatory drugs in the treatment of infections has great potential due to the possibility of use in both local and systemic therapy. However, preclinical and clinical phase studies will also have to determine a safe dose. Combination therapy with NSAIDs and antibiotics has demonstrated synergy and seems to be a good solution.

#### 2.3.2 Drugs used in general and local anesthesia

The drugs used in general and local anesthesia inhibit the activity of the nervous system by suppressing synaptic transmission; this is believed to result from the intensification of inhibitory processes, or suppression of stimulatory processes associated with such activity. Administration thus causes reversible loss of consciousness, amnesia, immobility and analgesia (Tesoro et al., 2020; Hogarth et al., 2023). Due to the interaction with γ-aminobutyric acid (GABA), N-methyl-D-aspartate (NMDA), glycine, glutamic acid, nicotinic acetylcholine receptors and, equally importantly, with voltage-dependent sodium, potassium, and calcium channels, it blocks the depolarization of the nerve cell membrane (Yang et al., 2024). In addition to their anesthetic effect, some drugs from this group, such as ketamine, propofol or lidocaine, have demonstrated *in vitro* anticancer activity by preventing cell proliferation and inducing apoptosis (Zhou et al., 2020; Wu et al., 2022). Research is also underway on the possibility of their use in the treatment of anorexia nervosa (NCT04714541) or postpartum depression (NCT03927378, NCT05907213).

Some anesthetic drugs also have antimicrobial activity. *In vitro*, studies have confirmed the effectiveness of ketamine against strains of *P. aeruginosa*, *E. faecalis*, *S. pyogenes*, *S. epidermidis* and *S. aureus*, including those resistant to methicillin (Begec et al., 2013; Coutinho et al., 2021; Sokolowska et al., 2023). The drug is believed to induce apoptosis by altering the integrity of the cell membrane and damaging bacterial DNA. It has also demonstrated affinity for sortase A, which is an important virulence factor in staphylococci. This enzyme is responsible for the acquisition of iron from host cells, the adhesion of bacteria to the extracellular matrix of the host, and the evasion of its immune system. A similar mechanism of antibacterial activity occurs in the case of lidocaine and procaine, these damage the bacterial cell membrane, leading to changes in permeability and ion outflow within the bacterial cell (Coutinho et al., 2021). An ongoing clinical trial (NCT04843982, in the recruitment phase) aims to evaluate the anti-inflammatory effect of one of the ketamine stereoisomers in the acute phase of sepsis. Research on the possibility of repositioning anesthetic drugs in the treatment of infectious diseases is currently in the preclinical phase.

**FIGURE 4:**
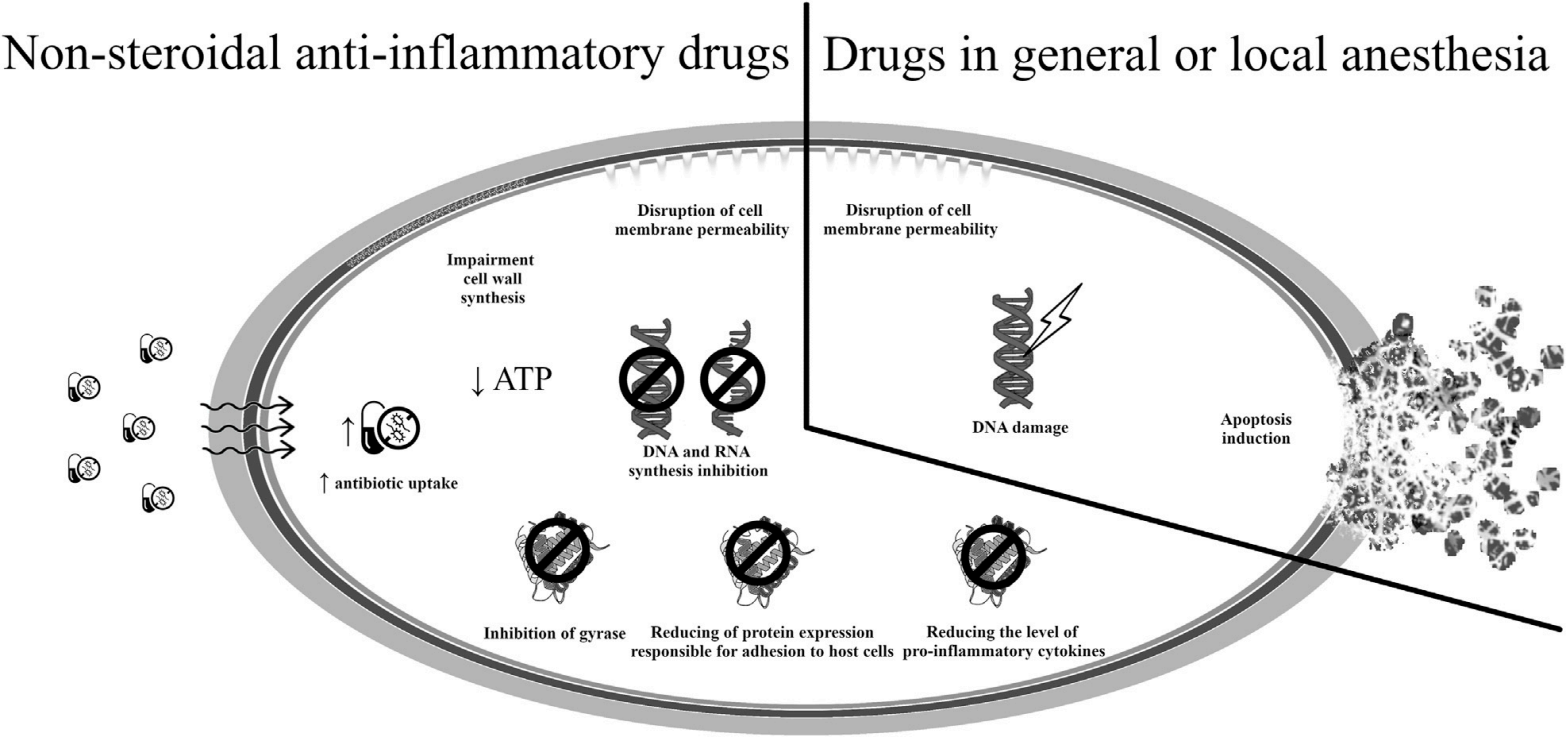
Potential mechanisms of antibacterial action of medicines used in therapy against pain.

The potential mechanisms of antimicrobial action of drugs used in pain therapy are presented in Figure 4 (Chiu et al., 2009; Begec et al., 2013; Maitre et al., 2022; Ferrer-Luque et al., 2023; Tabatabaeifar et al., 2023).

### 2.4 Medicines used to treat cardiovascular disorders

Cardiovascular diseases are often treated using β-adrenergic receptor antagonists and calcium channel blockers. β-adrenergic receptor antagonists (β-blockers) weaken the action of catecholamines, causing a decrease in chronotropy and inotropy (Pascual et al., 2016; Goldfine et al., 2023). The calcium channel blockers include dihydropyridines (e.g., amlodipine) and non-dihydropyridines (e.g., diltiazem or verapamil). Non-dihydropyridines are characterized by greater selectivity towards cardiac calcium channels. Dihydropyridines act on vascular smooth muscle cells, leading to their dilation; however, loss of selectivity may result in lower cardiac contractility (Goldfine et al., 2023). Medicines used to treat cardiovascular disorders are also being tested for their anticancer and anti-inflammatory potential (Qasim et al., 2020; Liang et al., 2021).

The calcium channel blocker amlodipine, in addition to its antihypertensive effect, has also shown antibacterial activity by inhibiting β-lactamases (Hua et al., 2022). Among the non-selective β-adrenergic receptor antagonists, carvedilol demonstrated synergistic effects when used in combination with gentamicin or with amlodipine against strains of the same species (Ugurel and Turgut-Balik, 2023).

Recent studies have shown that calcium channel blockers, such as lacidipine, nifedipine, and verapamil, could inhibit the growth of Gram-positive and Gram-negative microorganisms including *S. aureus* and *Vibrio cholerae* (Hua et al., 2022). Verapamil is an anti-hypertensive and antiarrythmic drug with a broad spectrum of antibacterial activity. Its effectiveness against *P. aeruginosa*, *S. aureus*, and *M. tuberculosis* strains has been demonstrated, as well as the ability to reduce the resistance of *S. aureus* strains to fluoroquinolones (Thangamani et al., 2015; Foletto et al., 2021a). Verapamil exerts an antituberculosis effect by inhibiting antibiotic efflux pumps and intensifying the action of drugs typically used in the treatment of tuberculosis; this may be an indication for combination therapy (Singh and Chibale, 2021). This enhanced effect has been demonstrated for rifampicin, isoniazid, ethambutol and fluoroquinolones (Rodrigues et al., 2020). Additionally, the molecule reduced the tolerance of both susceptible and drug-resistant mycobacterial strains to bedaquiline, clofazimine, and moxifloxacin (Adams et al., 2014; Andries et al., 2014). However, very high concentrations of verapamil are required to achieve therapeutic concentrations in *M. tuberculosis* infections, indicating that further research is needed on more effective and selective structural analogues (Singh et al., 2014; Padmapriyadarsini et al., 2023). It also showed antifungal activity against various *Candida* spp. and was effective in inhibiting infections caused by influenza A viruses, in which regulation of calcium concentration played a key role in inhibiting viral protein transport and maturation (Jayaseelan and Paramasivam, 2020; Scorzoni et al., 2020). Preclinical studies indicate that drugs used in cardiovascular diseases have a wide spectrum of antimicrobial activity; however further research is necessary to determine the appropriate doses of the drugs in these indications.

**FIGURE 5:**
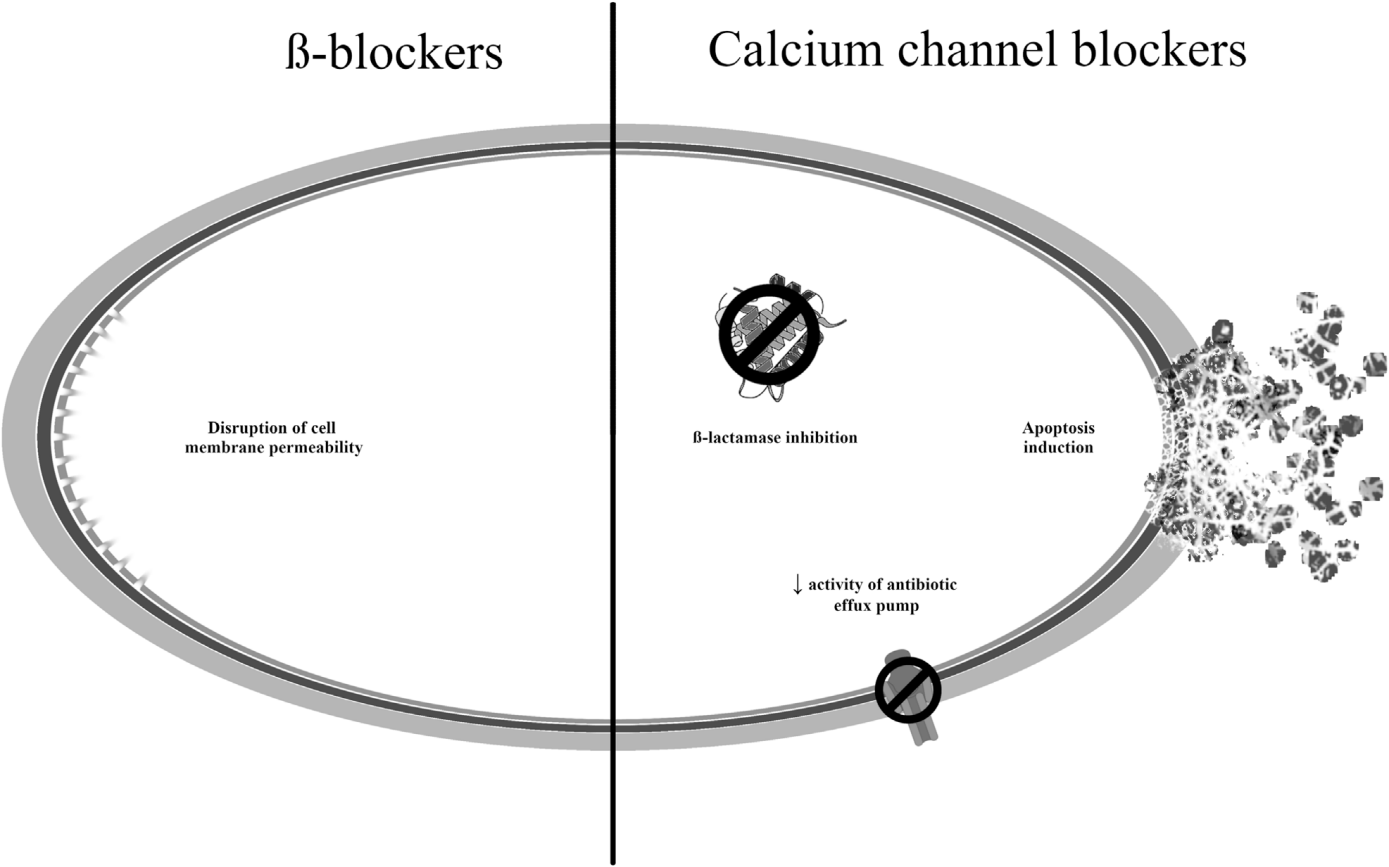
Potential mechanisms of antibacterial action of medicines used to treat cardiovascular disorders.

The mechanisms of antibacterial action of the drugs described in this section are presented in Figure 5 (Zawadzka et al., 2019; Singh and Chibale, 2021; Abdel-Karim et al., 2022; Hua et al., 2022; Barbosa et al., 2023).

### 2.5 Medicines used in neurological therapy and psychiatry

#### 2.5.1 Antipsychotics

The first generation of typical antipsychotic drugs, e.g., including haloperidol, fluphenazine, thioridazine and loxapine, were first used in the 1950s. The act by regulating the neurotransmitters in the brain, most important by blockading the D2 dopamine receptor (Weston-Green, 2022). Atypical antipsychotics, such as olanzapine or quetiapine, are characterized by high affinity for 5-HT2 receptors. Drugs in this group are considered capable of binding to multiple therapeutic targets, resulting in multiple mechanisms of action. They interact not only with neurotransmitter receptors, but also have the ability to suppress NF-kB, deregulate cyclins, or phosphorylate β-catenin (Awuah et al., 2023).

Antipsychotics are used to treat mental disorders and psychoses, especially schizophrenia and bipolar disorder (Vitiello et al., 2009; Lin et al., 2022). Research indicates the possibility of their use as complementary drugs in anticancer therapy, and this has been the subject of several clinical trials (Vlachos et al., 2021). The anticancer effect may result from modulation of various signaling pathways as well as autophagy, and influencing changes in cell membrane permeability and cell metabolism (Rácz and Spengler, 2023). For example, chlorpromazine and thioridazine have been included in clinical trials as an adjunct in cancer treatment, as well as in the treatment of migraine (NCT05190315, NCT05967442) (Aslostovar et al., 2018; Cohen and Friedman, 2021).

Among the antipsychotics, flupentixol, thioridazine, and prochlorperazine have demonstrated antibacterial activity. They may act by inhibiting the enzymes influencing the bacterial cell membrane and antibiotic efflux pumps (Barbarossa et al., 2022; Hua et al., 2022). It has been demonstrated that phenothiazines can reduce the ability of microorganisms to adhere to endothelial cells and can interfere with the action of ATPases, which inhibits bacterial replication (Barbarossa et al., 2022). Prochlorperazine was found to have antibacterial activity against Gram-negative and Gram-positive bacteria by infuencing the functioning of efflux pumps. Flupentixol, in turn, reduced the membrane potential in *S. aureus* cells (Hua et al., 2022). Importantly, a study on urinary catheters found chlorpromazine to be effective against the bacterial species most commonly implicated in urinary tract infections, i.e., *E. coli*, *P. mirabilis* and *Klebsiella* spp.; hence it may be suitable for coating urological instruments and preventing urinary tract infections (Sidrim et al., 2019).

Interestingly, thioridazine, withdrawn from treatment of schizophrenia due to cardiotoxicity, turned out to be effective against methicillin-sensitive and methicillin-resistant strains of *S. aureus*, *Enterococcus* spp., and *M. tuberculosis*, *M. avium* (Kristiansen et al., 2007; Deshpande et al., 2016; Ruth et al., 2020). Also, among the phenothiazine derivates, it also the most promising candidate for use as an antituberculosis drug; it inhibits the action of efflux pumps and increases the killing activity of macrophages. The antimycobacterial effect was associated with the inhibition of potassium and calcium channels, which led to a decrease in pH in the phagolysosome, activation of hydrolases, and subsequent destruction of *M. tuberculosis* cells (Rodrigues et al., 2020). The levorotatory form showed better antibacterial activity, both *in vitro* and *in vivo*; racemic thioridazine was found to reduce the ability of Gram-positive and Gram-negative bacteria to invade cell lines (Foletto et al., 2021a). However, care should be taken to minimize its negative effects on the central nervous system and cardiotoxicity. Hence, there is currently considerable interest in identifying suitable thioridazine analogues or changing the form of the drug, e.g., encapsulation of the drug in nanoparticles (Rodrigues et al., 2020). Currently, research on the repositioning of antipsychotic drugs in the treatment of infectious diseases is in the preclinical phase.

#### 2.5.2 Antidepressants

The most commonly prescribed antidepressants include selective serotonin reuptake inhibitors (SSRIs) and tricyclic antidepressants (TCAs). They improve mood and anxiety disorders by inhibiting the transporters responsible for the reuptake of norepinephrine and serotonin. Currently, antidepressants are typically used to treat neuropathic pain in oncological patients. Studies in animal models indicate their analgesic effect is associated with their potential to achieve high noradrenaline concentrations through their action on α2-adrenergic receptors. Dopamine and serotonin may also enhance the analgesic effects of norepinephrine. Additionally, TCAs also inhibit the production of the pro-inflammatory cytokines TNF-α, IL-1β, and IL-6 (Asensi-Cantó et al., 2022; Rácz and Spengler, 2023). They can also inhibit cancer cell growth by blocking their molecular pathways. These phenomena support the rationale for further trials on the usage of TCAs in cancer diseases.

Antidepressants, particularly serotonin reuptake inhibitors (SSRIs), have also been recognized as potential candidates for drug repurposing for treating infectious diseases. For example, sertraline has been demonstrated to enhance the activity of fluoroquinolones and aminoglycosides against *S. aureus* strains, and its antimicrobial effect in combination with gentamicin and erythromycin, has also been evaluated against *P. aeruginosa* and *E. coli*. Sertraline has also shown antibacterial activity against *Helicobacter pylori* strains in a concentration-dependent manner, as well as possible synergism with amoxicillin, clarithromycin, tetracycline, and metronidazole, used to eradicate *H. pylori*. The potential mechanism could involve the inhibition of bacterial protein translation and interference with bacterial efflux pumps (Barbarossa et al., 2022). In another study, sertraline increased the effectiveness of polymyxin B against strains of other Gram-negative species such as *A. baumannii*, *E. coli*, and *K. pneumoniae* (Otto et al., 2019), as well as against strains of *Enterococcus* spp. resistant to vancomycin (Foletto et al., 2021a). The bactericidal effect of sertraline against the reference strains *S. aureus*, *E. faecalis*, *B. cereus*, and *E. coli* was found to be further enhanced by combining it with disulfiram, used in the treatment of alcoholism. Disulfiram was metabolized by bacterial cells to diethyldithiocarbamate (DDTC), which has antibacterial properties. Additionally, disulfiram has a proteolytic effect due to its ability to chelate ions (Serafin et al., 2020). Clinical trials also included combination therapy of fluconazole and sertraline in the treatment of early disseminated cryptococcal infection (NCT03002012). However, when high doses of sertraline were used, the clinical trial was terminated due to high rate of adverse events (Boulware et al., 2020). *In vitro* studies have demonstrated synergistic and additive effects of SSRIs in combination with azoles. Sertraline had the best antifungal activity against *Cryptococcus* spp.; it was believed to act by damaging the mitochondrial membrane and increasing the production of reactive oxygen species, leading to apoptosis (da Silva et al., 2023).

In turn, fluoxetine showed activity against MRSA strains, vancomycin-resistant *Enterococcus* spp. strains, and *A. baumannii* strains, which was probably caused by changes in the integrity of bacterial plasma membranes and damage to their DNA, leading to bacterial cell death (Foletto et al., 2020; Foletto et al., 2021a; Foletto et al., 2021b). Both fluoxetine and paroxetine showed improved efficacy in combination with ciprofloxacin against both Gram-negative and Gram-positive microorganisms, compared to ciprofloxacin alone. When the antidepressant was combined with this fluoroquinolone, its potency against MDR-resistant strains of *E. coli*, *A. baumannii*, *K. pneumoniae*, and *E. faecium* was eightfold increased (Foletto et al., 2020; Foletto et al., 2021b). SSRIs can affect a number of processes regulating the biosynthesis of products important for microorganisms, regardless of absorption into bacterial cells. The mechanism of action may also be based on the inhibition of bacterial efflux pumps (de Sousa et al., 2018; Foletto et al., 2020; Foletto et al., 2021a) or on disturbing bacterial cell wall synthesis and preventing bacterial cell division (Basha et al., 2018). Moreover, fluoxetine affected plasma membrane exopolysaccharides integrity and induced DNA damage, which may lead to bacterial cell apoptosis (Neto et al., 2019; Foletto et al., 2021b).

In turn, amitriptyline, a TCA, had antibacterial activity against carbapenemase-producing strains of *K. pneumoniae*. A synergistic effect was also obtained when combined with colistin, i.e., the “last resort” antibiotic, and tetracyclines (Barbarossa et al., 2022; Ugurel and Turgut-Balik, 2023).

#### 2.5.3 Medicines used in epilepsy

Valproic acid, used in the treatment of epilepsy and bipolar disorders, stimulates the formation of autophagosomes *in vitro*. Its has been found to enhance the clinical effects of isoniazid and rifampicin in the treatment of tuberculosis, as demonstrated in studies on human cell lines. The antimycobacterial activity of valproic acid may result from the ability to inhibit succinic semialdehyde dehydrogenase, associated with the process of aerobic respiration. Valproic acid also influences the metabolism of fats, thus regulating the fluidity of cell membranes, and reduce the amount of substrate for the production of prostaglandins (Rodrigues et al., 2020). It is currently also the subject of several clinical trials regarding its effectiveness in the treatment of neuropathic pain or anticancer therapy (NCT01928849) (Caponigro et al., 2016; Iannelli et al., 2020).

#### 2.5.4 Medicines used in neurodegenerative diseases

Entacapone and tolcapone are able to reversibly inhibit catechol-O-methyltransferase (COMT), and are hence used in the supportive treatment of Parkinson’s disease. These drugs have also shown antituberculosis activity (Maitra et al., 2015; Beklen et al., 2021). The possible mechanism of action against *M. tuberculosis* strains might be based on their ability to inhibit the synthesis of mycolic acid: a compound necessary for the construction of the mycobacterial cell wall. Its loss leads to the death of bacterial cells (Maitra et al., 2015; Sharma et al., 2023). Unlike isoniazid, entacapone and tolcapone do not require enzymatic activation, so there is no possibility of mutations that can develop resistance (Maitra et al., 2015).

Memantine, a drug used to treat Alzheimer’s disease, has also shown antibacterial properties. It acts by inhibiting N-methyl-d-aspartate (NMDA) receptors in the central nervous system (Johnson and Kotermanski, 2006). Moreover, the discovery of NMDA receptors in non-neuronal tissues, such as the heart, lungs, and kidneys, suggests it may have value across a broader therapeutic spectrum (Zakariaa et al., 2023). Memantine, used in combination with ampicillin, blocked the inflammatory response to bacterial infection caused by *E. coli*. Studies indicate that memantine affects the α7 nAChR receptor: an inflammation regulator also controlled by bacterial cells. It also reduces the intracellular survival of bacteria, both during bacteremia and in the later phase of meningitis caused by *E. coli*. Memantine may make it more difficult for the pathogen to penetrate the blood-brain barrier, as evidenced by studies in animal models. Its ability to inhibit the development of meningitis is correlated with the degree of bacteremia development. The proposed doses of this drug showed neuroprotective properties and did not have a toxic effect on human cells (Yu et al., 2015).

**FIGURE 6:**
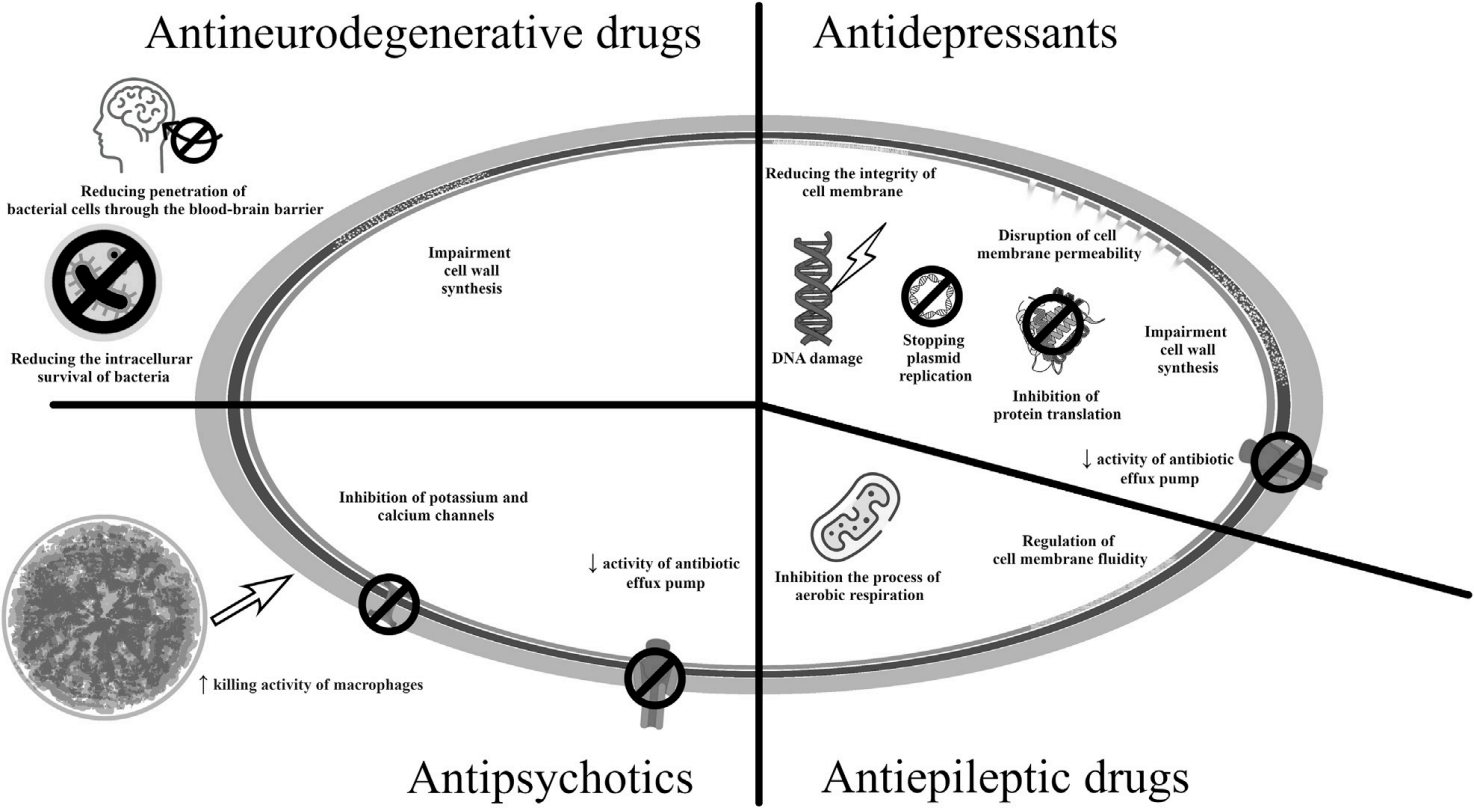
Potential mechanisms of antibacterial action of medicines used in neurological therapy and psychiatry.

The mechanisms of action of antibacterial drugs used in neurological diseases and psychiatry are presented in Figure 6 (Johnson and Kotermanski, 2006; Marchi et al., 2015; Caponigro et al., 2016; Basha et al., 2018; de Sousa et al., 2018; Saputo et al., 2018; Neto et al., 2019; Foletto et al., 2020; Beklen et al., 2021; Maitre et al., 2022).

### 2.6 Medicines used in parasitic infections

Antiprotozoal drugs have also demonstrated antibacterial effects. One of them is pentamidine, which disrupts cellular metabolism and affects the metabolism of nucleic acids, folic acid and proteins. Pentamidine has been proven to treat infections caused by multidrug-resistant Gram-negative bacilli by affecting the integrity of the outer bacterial membrane (Hua et al., 2022).

Despite being currently used as an anthelmintic drug in veterinary medicine, salicylanilide derivatives have also shown good effects in antimicrobial therapy. Among these, niclosamide is used to treat tapeworm and roundworm infections and has been widely studied as an antimicrobial drug. It has demonstrated high activity against strains of Gram-positive bacteria, MRSA, and vancomycin-resistant *E. faecium* (Domalaon et al., 2019). Niclosamide is also considered as a candidate for repositioning in cancer; it has been shown to inhibit the Wnt/β-catenin pathway, which is important for embryogenesis and cell differentiation, and is involved in the proliferation of cancer tissue. However, it is difficult to maintain the concentrations of the drug within the therapeutic range (Schweizer et al., 2018). Niclosamide disrupt the proton flow within the cell membrane, which also inhibits the growth of *H. pylori*. The drug has demonstrated synergistic effects with colistin in the case of resistant strains *A. baumannii* and *K. pneumoniae* (Barbarossa et al., 2022).

Colistin has also displayed synergy with the salicylanilide derivates oxyclozanide, rafoxanide, and closantel against multidrug-resistant strains of *P. aeruginosa*, *A. baumannii*, *K. pneumoniae*, *E. coli*, and *E. cloacae*. The development of derivatives with better pharmacokinetic properties and a better toxicity profile could influence the effectiveness of therapy for infections caused by multidrug-resistant Gram-negative bacilli (Tran et al., 2016; Domalaon et al., 2019).

Ivermectin has antiparasitic activity due to its ability to activate a glutamate-gated chloride channel, which does not occur in vertebrate organisms. It is effective against nematodes and arthropods (Hu et al., 2023). In vertebrates, it has the ability to inhibit type A γ-aminobutyric acid and glycine receptors and activate the acetylcholine receptor in brain nerve cells. Because of its mechanism of action, ivermectin is being investigated for its repurposing in the treatment of disorders associated with alcohol abuse or epilepsy. However, a significant problem is the poor ability of this molecule to penetrate the blood-brain barrier (Loescher, 2023). The justification for its use is based on the proven anti-inflammatory effect due to the inhibition of pro-inflammatory cytokines, and antiparasitic effect on demodex (Baranska-Rybak and Kowalska-Oledzka, 2019). Ivermectin is also among the antiparasitic drugs with antibacterial properties. *In vitro*, its activity has been demonstrated against *S. aureus* strains and the biofilm they create (Ashraf et al., 2018). Ivermectin itself has a bacteriostatic effect, while its analogues may have a bactericidal effect. It is indicated that structural analogues destroy the bacterial cell wall, additionally interact with cell membranes and influence their permeability. Moreover, they have very good anti-biofilm properties against MRSA (Tan et al., 2021). Among antiparasitic drugs, thiabendazole, as a representative of benzimidazoles, also showed antitubercular activity. This drug has the ability to inhibit *M. tuberculosis* cell division. It is also indicated that this compound has the ability to inhibit the enzymes succinate dehydrogenase and fumarate reductase, which are involved in the aerobic respiration of tuberculous mycobacteria (Rodrigues et al., 2020).

**FIGURE 7:**
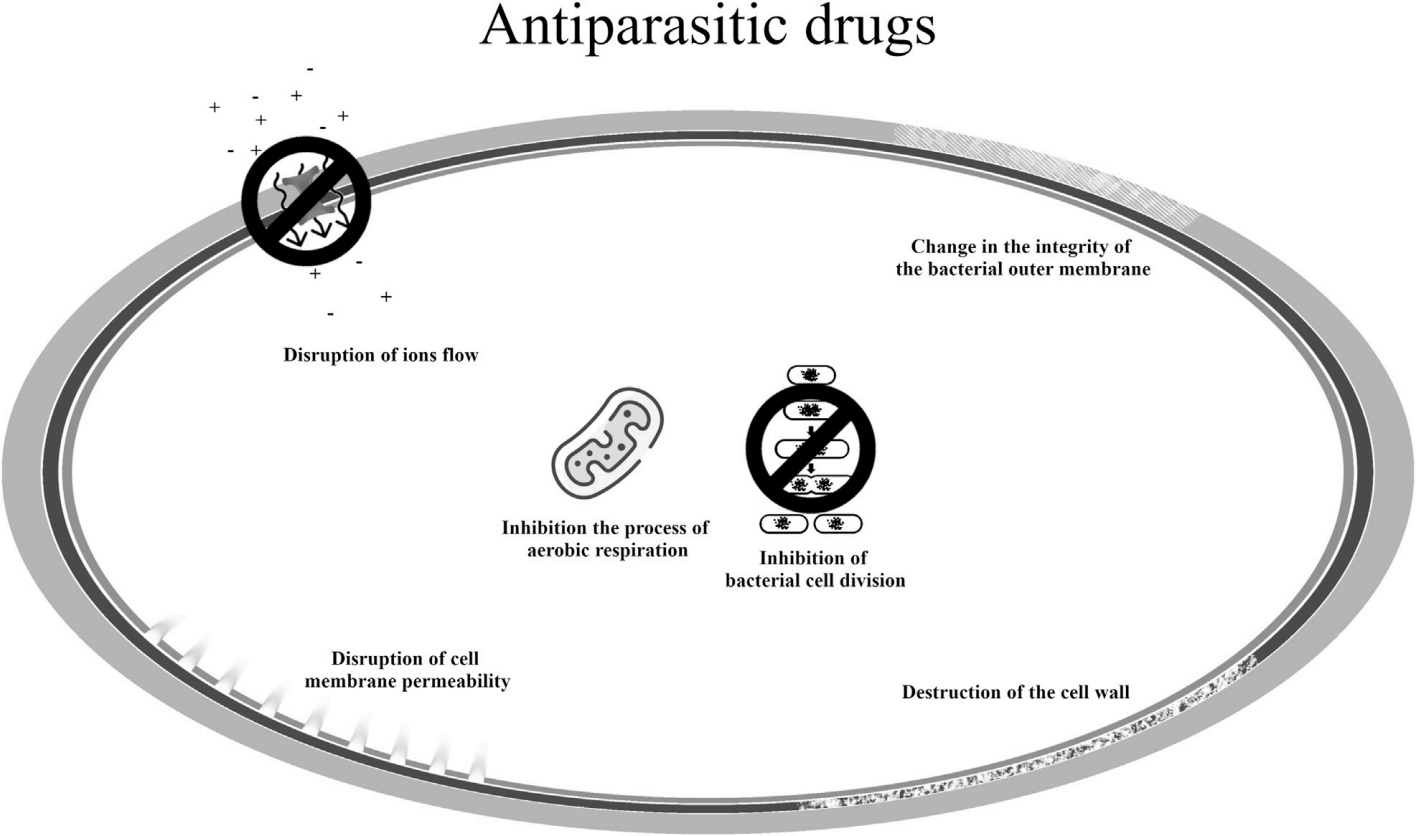
Potential mechanisms of antibacterial action of medicines used in parasitic infections.

The mechanisms of action of antiparasitic drugs in the new indication are summarized in Figure 7 (Rodrigues et al., 2020; Tan et al., 2021; Hua et al., 2022; Hu et al., 2023).

### 2.7 Medicines used in metabolic disorders

#### 2.7.1 Antidiabetic drugs

One of the antidiabetic drugs that inhibit the growth of bacteria is metformin, for which the possibility of repositioning is being investigated in many indications (Gadducci et al., 2016; Matsuoka et al., 2021; Agostini et al., 2022). While metformin has remained the first-line medication to treat type 2 diabetes mellitus (T2DM), its mechanisms of action are complex and not fully understood. The molecule has been recognized to act through activated protein kinase (AMPK)-dependent and AMPK-independent mechanisms in the liver, where it controls hepatic glucose production. The current understanding of the molecular mechanisms of metformin action focuses on the other sites of its pharmacological activity, and consequently, recent years have brought an increasing interest to repurpose metformin for the treatment of cancer, age-related diseases, inflammatory diseases or COVID-19 (Foretz et al., 2023). Metformin has also been successfully repositioned in the treatment of polycystic ovary syndrome.

The use of metformin and Triton X-100 allowed for a reduction in cell viability and a reduction in the virulence potential of *E. faecalis* (Barbarossa et al., 2022). It has been suggested that the antibacterial effect may be related to the rupture of the inner bacterial membrane (Foletto et al., 2021a). *In vivo* studies also found that metformin increases phagocytosis by macrophages in the case of *E. coli* infection and reduces the severity of the disease in the case of *M. tuberculosis* infection (Brown, 2015). It has also been shown that in the case of tuberculosis, the use of metformin inhibits cell necrosis and contributes to tissue regeneration (Guler and Brombacher, 2015). Metformin also promotes autophagy in infected macrophages and limits the development of mycobacteria by positively influencing the production of reactive oxygen species (Vyas et al., 2022). For this reason, the molecule is currently in clinical trials for the treatment of tuberculosis (NCT05215990, NCT04930744). These are phase 1 and phase 2 interventional studies aimed at assessing whether metformin will have an impact on lung damage and the duration and effectiveness of therapy in tuberculosis. Other studies are ongoing to assess the effect of metformin on mortality in patients with and without diabetes and on inflammatory markers in sepsis and septic shock (NCT05572060, pre-recruitment; NCT06181422; pre-recruitment; NCT05979038, in the recruitment phase—phase 2 and 3 clinical trials).

Acarbose, which inhibits α-glucosidase activity in the small intestine, also has many additional effects beyond its effectiveness in diabetes. *In vitro* studies have shown that it reduces the inflammation, oxidative stress and platelet activation observed in the course of atherosclerosis (Chan et al., 2016). In *in vitro* studies, acarbose showed activity against *M. tuberculosis*: its addition allowed the doses of isoniazid and ethambutol to be halved (Sharma et al., 2023). Additionally, at high concentrations, acarbose was able to inhibit biofilm formation by *M. tuberculosis* when combined with ethambutol and isoniazid (Kumar et al., 2019b). Similarly, it was found to be effective against respiratory infections caused by *P. aeruginosa* strains in a type 2 *in vitro* diabetes model; treatment anti-inflammatory and antimicrobial effects and reduced mortality (Liu et al., 2023).

#### 2.7.2 Hypolipemic drugs

Statins are a group of drugs used in the treatment of hypercholesterolemia and in the prevention of atherosclerosis. They are responsible for inhibiting the enzyme hydroxy-3-methylglutaryl coenzyme A (HMG-CoA) reductase which is involved in the synthesis of cholesterol in the liver (Foletto et al., 2021a). In recent years, HMG-CoA reductase inhibitors (HMGRI, statins) have emerged as the most important class of lipid-lowering agents. Clinical trials have confirmed the beneficial effects of statins in cardiovascular disorders, in primary and secondary prevention settings, and in asymptomatic subjects with a high cardiovascular risk (Jasinska et al., 2007; Jiang et al., 2021). They are prescribed, among others, after myocardial infarction and ischemic stroke, and in the treatment of atherosclerosis or coronary artery diseases (Murphy et al., 2020). It is well-known that statin pleiotropy provides various beneficial effects in addition to their lipid-lowering properties, such as improved endothelial dysfunction and better nitric oxide bioavailability; treatment also has antioxidant effects, anti-inflammatory and immunomodulatory properties, and has been found to stabilize atherosclerotic plaques and inhibit cardiac hypertrophy. In addition, statins have demonstrated anti-tumor properties, which have attracted particular attention for repurposing (Jasinska et al., 2007; Rohilla et al., 2016; Jiang et al., 2021). Some studies have shown HMGRI to have potential efficacy in dementia and Alzheimer’s disease, non-alcoholic fatty liver disease, and due to their immunomodulatory and antioxidant properties, also in rheumatoid arthritis (Murphy et al., 2020; Aminifar et al., 2023).

A wealth of evidence shows that people taking statins could have a lower risk of bacterial infections and better survival during infection. They are less exposed to community-acquired blood infections caused by *S. aureus* strains. Additionally, the risk of developing sepsis was also lower. *In vivo* studies have shown that simvastatin treatment is beneficial in lung infections caused by *S. aureus* strains: it lowered inflammatory marker levels and reduced mortality in animal models (Evans and McDowell, 2021). It has been proposed that the drugs exert their antibacterial activity by promoting apoptosis, and the mechanism of action is unrelated to the inhibition of HMG-CoA reductase (Foletto et al., 2021a).

Antibacterial effects were also demonstrated by atorvastatin and pitavastatin. It has been proposed that they act by breaking down the structures of teichoic acid and reducing the number of alanine residues on the surface of Gram-positive bacteria cells, which would reduce the ability of bacterial cells to form a biofilm (Barbarossa et al., 2022). Atorvastatin, lovastatin, and simvastatin have demonstrated significant inhibition of bacterial growth in biofilms, including *S. mutans* (Saputo et al., 2018).

Simvastatin also showed synergy of action with silver ions against MRSA strains and against *E. coli* producing β-lactamases with an extended substrate spectrum. Treatment resulted in distorted and lysed bacterial cells associated with a change in membrane permeability (Barbarossa et al., 2022). In an animal model of diabetes, simvastatin also accelerated wound healing and angiogenesis, influencing the control of inflammation by limiting the production of TNF-α and IL-6. Therefore, it may be an adjunct drug in the treatment of skin infections caused by MRSA strains (Thangamani et al., 2015). Atorvastatin was similarly active against both Gram-positive bacteria (*S. aureus*) and Gram-negative bacteria (*A. baumannii*, *E. aerogenes*, *E. coli*) (Foletto et al., 2021a). Atorvastatin is currently involved in clinical trials for the treatment of bronchiectasis (NCT01299194), a chronic lung disease characterized by thick secretions and frequent respiratory infections. In most patients, it leads to chronic bacterial colonization.

*M. tuberculosis* can use cholesterol contained in host macrophages for infection and its own survival (Su et al., 2021). Therefore, the presence of higher cholesterol levels may be a predisposing factor to tuberculosis, and reducing its level may help limit the entry of bacteria to macrophages. Simvastatin, in combination with rifampicin, isoniazid, and pyrazinamide, has been shown to be effective against infections caused by *M. tuberculosis*. These combinations show increased mycobacterial killing, a decrease in the number of colony-forming units in the lungs, and a shorter time to obtain a pure culture. Additionally, the inflammatory process was also found to be regulated by increased secretion of the cytokines IL-12, IL-1β, and IL-10 (Sharma et al., 2023). *In vivo* models have shown that statins shorten the cure time for tuberculosis and have a beneficial effect on changes in the lungs. They also have the ability to inhibit T cell activation induced by *M. tuberculosis* antigens. Additionally, their use significantly reduces the rate of tuberculosis recurrence by almost 50%. As a consequence, several clinical trials are currently being conducted on the effectiveness of statins as an adjuvant therapy in tuberculosis (NCT04504851, NCT04721795, completed, no trial results; NCT06199921, in the recruitment phase). Atorvastatin is also being considered for treatment to reduce inflammation after TB treatment (NCT04147286, in the recruitment phase). These clinical trials on the use of statins in the treatment of tuberculosis are currently in phase 2 and 3 of clinical trials.

**FIGURE 8:**
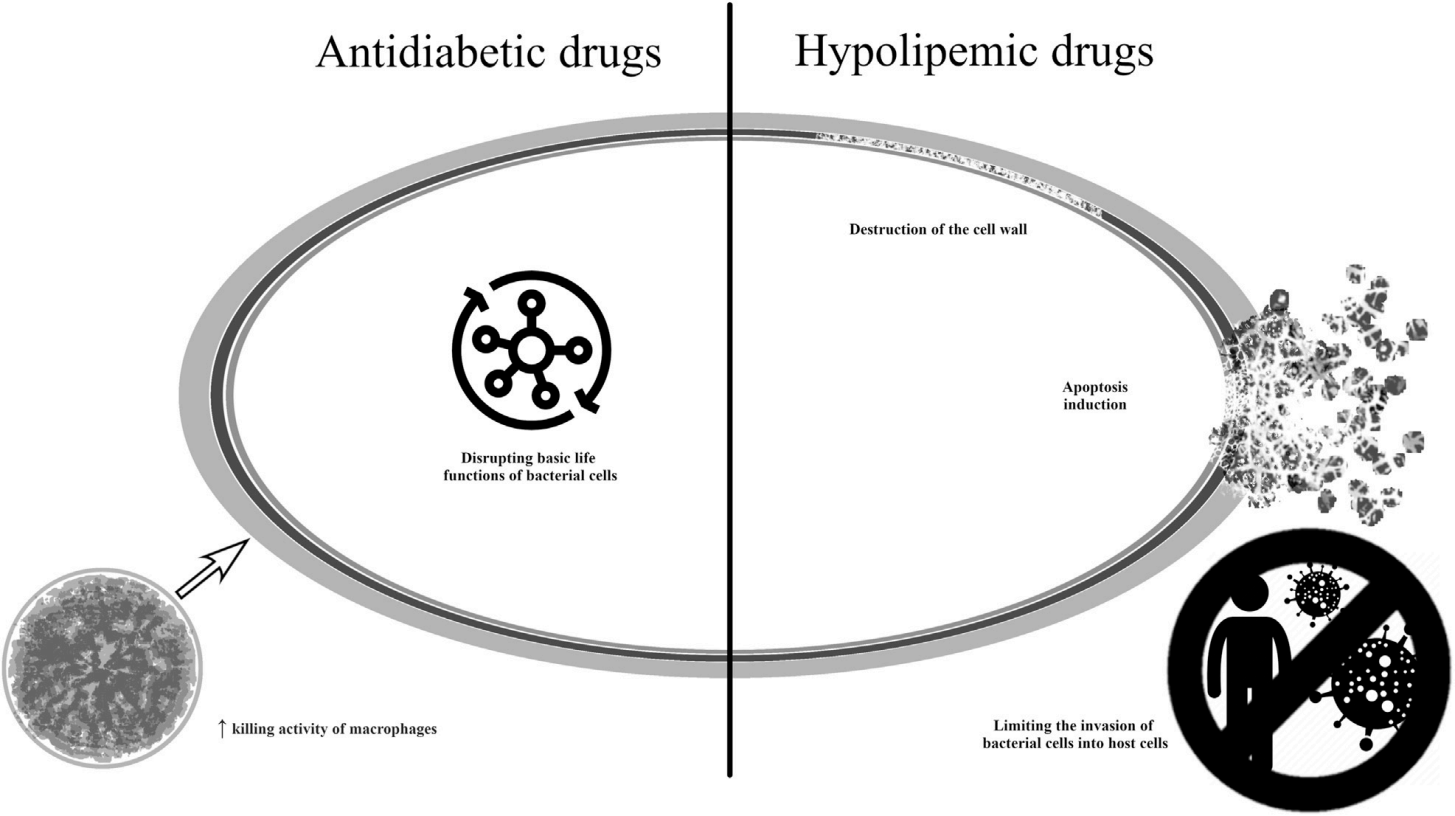
Potential mechanisms of antibacterial action of medicines used in metabolic disorders.

The antibacterial mechanism of antidiabetic anh hypolipemic drugs is shown in Figure 8 (Brown, 2015; Brindha et al., 2016; Guerra-De-Blas et al., 2019; Evans and McDowell, 2021; Vyas et al., 2022).

### 2.8 Other examples of non-antimicrobial drugs with antimicrobial activity

Among drugs used externally, ciclopirox has antibacterial effect. It inhibited the growth of *A. baumannii*, *E. coli*, and *K. pneumoniae*, regardless of any antibiotic resistance of the strains. The probable mechanism of action results from the possibility of inhibiting the synthesis of lipopolysaccharide in Gram-negative bacteria, a structure that protects bacteria against various substances, including antibiotics. Hence, treatment could sensitize gram-negative bacteria to some groups of antibiotics used in infections with Gram-positive bacteria (Brown, 2015). Cyclopirox is currently used only topically, but due to new potential applications, including in the treatment of cancer, research is underway on the possibility of oral use (Weir et al., 2011). Among the drugs used externally, zinc pyrithione, an antiseborrheic substance, has demonstrated bacteriostatic properties against streptococci and staphylococci. Its antibacterial effect is believed to be related to the ability to chelate metals and transport them through bacterial membranes (Saputo et al., 2018).

Ebselen is an organoselenium compound studied for its antioxidant, anti-inflammatory, and cytoprotective properties (Maslanka and Mucha, 2023). This molecule displayed high antibacterial activity against strains of Gram-positive bacteria, including *S. aureus* resistant to methicillin, vancomycin, and linezolid. Additionally, it also increased survival in cases of sepsis in animal models. Ebselen also demonstrated activity against strains of vancomycin-resistant enterococci (VRE) and streptococci (Younis et al., 2015; Maslanka and Mucha, 2023). Ebselen also inhibited the production of toxins, α-hemolysin, and Panton-Valentine leukocidin, important virulence factors in the pathogenesis of *S. aureus*. It also reduced the survival of *S. aureus* and *E. faecium* bacterial cells in biofilms. When used topically, ebselen reduced the level of pro-inflammatory cytokines in skin infections caused by strains of *Staphylococcus*. It also showed synergistic activity in combination with many antibiotics, linezolid, clindamycin, vancomycin, chloramphenicol, erythromycin, rifampicin, and gentamicin (Maslanka and Mucha, 2023). The combination of ebselen with isoniazid could also bring good therapeutic effects also against antituberculosis drug-resistant strains of *M. tuberculosis* (Padiadpu et al., 2016; Sharma et al., 2023).

Ebselen inhibits bacterial thioredoxin reductase; this prevents the bacteria from reducing disulfides in many substrates, thus disrupting the synthesis of DNA and cellular proteins and inducing oxidative stress inside bacterial cells (Maslanka and Mucha, 2023). In intestinal infections, ebselen also has the ability to protect human cells against *Clostridioides difficile* toxins, which are responsible for the destruction of human intestinal cells and tissue damage. In such cases, ebselen treatment promotes better regeneration of the intestinal microbiome and reduces the risk of repeated infections (Bender et al., 2015; Garland et al., 2020).

Much of the repositioning research in this area has focused on auranofin, which has been used for several decades to treat rheumatoid arthritis. Studies have shown the drug to have bactericidal activity against *N. gonorrhoeae* strains, without any activity against commensal lactic acid bacteria. It could be an effective response to the growing resistance demonstrated by *N. gonorrhoeae* strains to recommended antibiotics. Importantly, it has been found to inhibit the secretion of the pro-inflammatory cytokine IL-8 by cervical cells and have a long-lasting post-antibiotic effect. Auranofin has also exhibited antibacterial activity against MRSA and VRE strains, and *C. difficile* causing ulcerative colitis (Elkashif and Seleem, 2020).

Auranofin has demonstrated weak activity against gram-negative bacteria, which may be due to the inability of the drug to penetrate the outer cell membrane. However, the combination of auranofin with colistin, which has permeabilizing properties, causes strong activity against Gram-negative bacterial cells, including *P. aeruginosa*, that form a biofilm (Torres et al., 2018). Additionally, good effects against clinical isolates of *K. pneumoniae*, *A. baumannii*, *P. aeruginosa*, *C. freundii*, *E. cloacae*, and *E. coli* with MDR resistance were obtained by the combination colistin-ceftazidime-auranofin and colistin-rifabutin-auranofin. It has been shown that adding a third drug to combination therapy with antibiotics may be beneficial in the treatment of infections with multidrug-resistant strains. Auranofin was also active against *S. pneumoniae* strains with MDR-type resistance (Sun et al., 2016).

Auranofin has recently been granted orphan drug status by the FDA for the treatment of amoebiasis in humans. This confirms the importance of drug repurposing also in the treatment of infections (Younis et al., 2015). Auranofin was also tested in HIV-1 eradication (NCT02961829). It was well tolerated in combination with antiviral drugs, and no serious adverse events were detected during the clinical study. This drug affected the viral reservoir by reducing the total viral DNA in blood cells (Diaz et al., 2019). The use of auranofin is also being considered as adjunctive therapy for lung cancer (NCT01737502) and giardiasis (NCT02736968).

The immunosuppressive drug cyclosporine A demonstrated antimicrobial activity against *M. tuberculosis*. It inhibited biofilm formation by *M. tuberculosis* cells and a synergistic effect when combined with ethambutol and isoniazid. Its role in bacterial activation in latent tuberculosis has also been analyzed: administartion was found to improve the availability of bacterial cells for anti-tuberculosis drugs, increasing their effectiveness. The use of a minimal concentration of cyclosporine A that inhibits the growth of *M. tuberculosis* strains in combination with anti-tuberculosis drugs could minimize its immunosuppressive effect (Kumar et al., 2019b). In addition to preventing transplant rejection, the clinical range of cyclosporine has been expanded to include autoimmune diseases such as severe rheumatoid arthritis, psoriasis, nephrotic syndrome, severe atopic dermatitis and uveitis. Cyclosporine is used to treat many eye diseases, e.g., dry eye syndrome, posterior blepharitis, spring and atopic keratoconjunctivitis. It has also demonstrated therapeutic effects against ulcerative colitis. In recent years, the drug has been found to possess special cardioprotective and neuroprotective properties in *inter alia* myocardial infarction, traumatic brain injury or stroke. However, the use of cyclosporine in these indications involves the use of high doses (Guada et al., 2016).

As mentioned earlier, disulfiram has also been shown to have antibacterial activity against strains of *M. tuberculosis*, including strains with MDR and XDR resistance types, as well as against non-tuberculous mycobacteria *M. fortuitum* and *M. abscessus* (Maitra et al., 2015; Saputo et al., 2018; Das et al., 2019). These species of nontuberculous mycobacteria are most often isolated from cases of lung, skin, or lymphatic system infections in immunocompetent people and may also cause disseminated infections in immunocompromised people (Gharbi et al., 2021). It has demonstrated synergy with drugs used in standard therapy, and reduced the bacterial load in macrophages more effectively than amikacin; it also minimized the number of mycobacterial cells in the kidneys in animal models of neutropenic bacteremia (Das et al., 2019).

**FIGURE 9:**
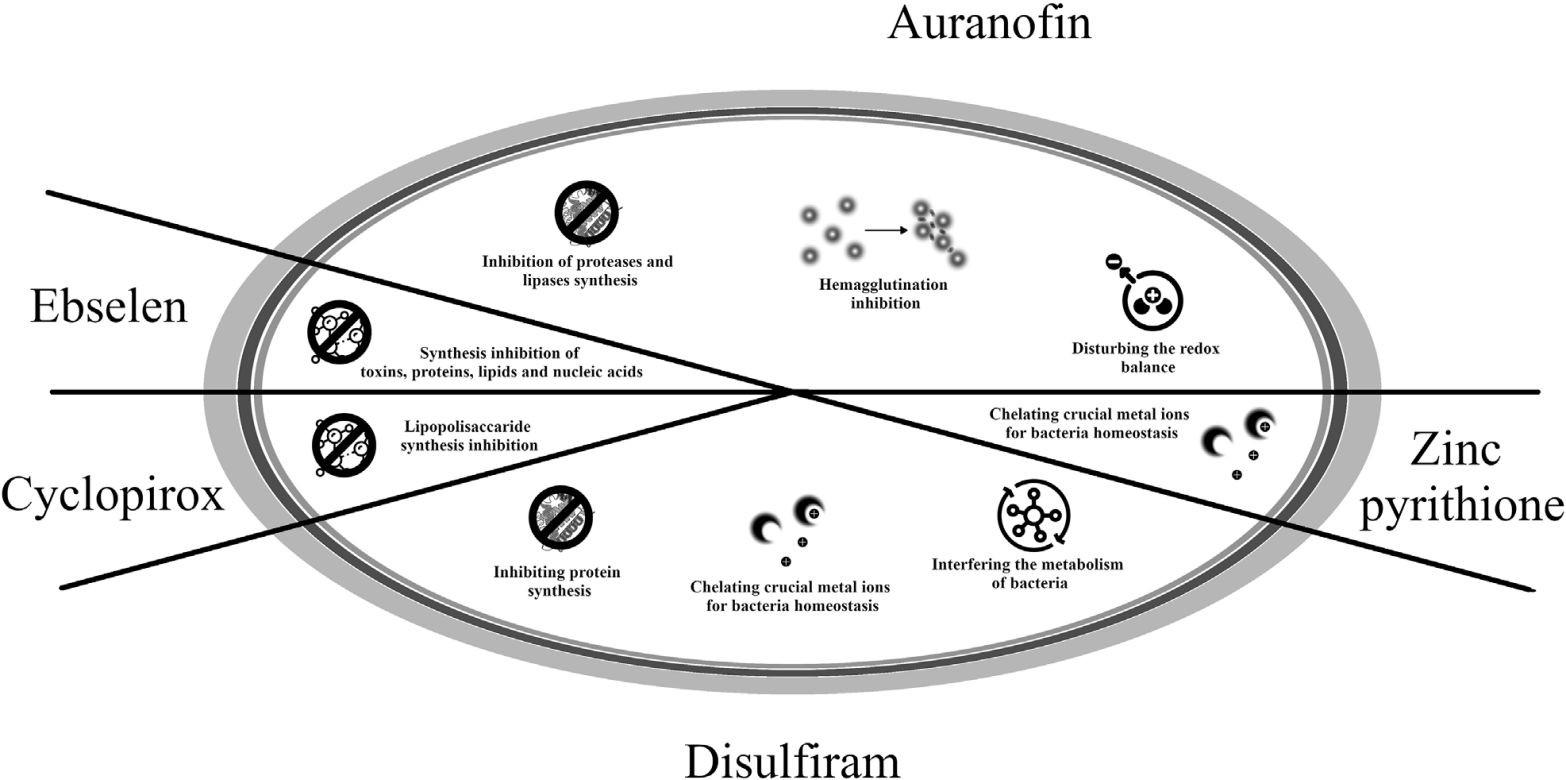
Potential mechanisms of action of repositioned drug candidates in infectious diseases—ciclopirox, zinc pyrithione, ebselen, auranofin and disulfiram.

The mechanisms of action of the drugs presented in this section are presented in Figure 9 (Younis et al., 2015; Saputo et al., 2018; Abutaleb and Seleem, 2020; Feng et al., 2021; Kobatake et al., 2021; Barbarossa et al., 2022; Maslanka and Mucha, 2023).

## 3 Future implications and limitations

Increasing antibiotic resistance among both Gram-negative and Gram-positive bacteria has prompted the search for new antimicrobial compounds. One direction of research involves repositioning drugs with known safety profiles. The data on the number of publications presented in Figure 1 indicate that increasing attention is being paid to the potential of drug repositioning in infectious diseases.

**FIGURE 10:**
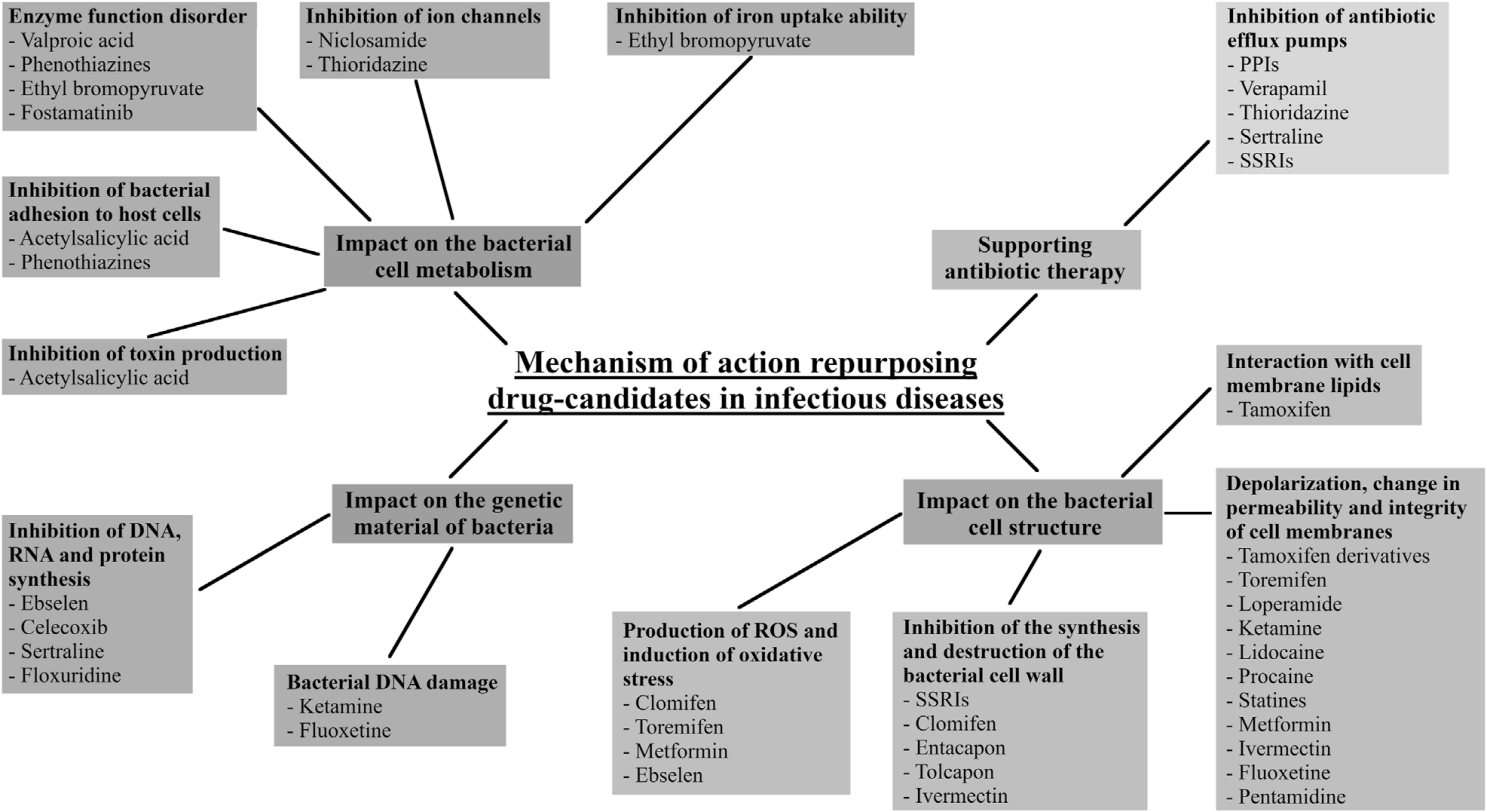
Mechanisms of action repurposing drug-candidates in infectious diseases.

Figure 10 summarizes and presents the mechanisms of action of repositioning drug candidates in infectious diseases. Most of the drugs discussed in this review cause structural changes within the bacterial cell and its metabolism; when used as combined therapy, an important mechanism of their action also involves inhibiting bacterial efflux pumps for antibiotics. Some of the drugs presented in this work, such as the SSRIs, show several mechanisms of action.

Many drugs used to treat diseases other than infections have antibacterial properties and synergism with antibiotics. In the face of the growing number of multidrug-resistant strains, combination therapy may prove to be the most effective solution, reduce the risk of antibiotic resistance and be the answer to the problem of antibiotic resistance. One of the important discoveries in this area is the combination of β-lactam antibiotics with non-antibiotic β-lactamase inhibitors, e.g., clavulanic acid, tazobactam or vaborbactam and relebactam, discovered in recent years. Additionally, the synergistic combination may extend the usefulness of well-established antibiotics in therapy. Table 1 summarizes the synergistic pairs of repositioning drug candidates and antimicrobial drugs presented in this manuscript.

**TABLE 1:** Synergistic combinations of candidates for new antibacterial drugs with existing antimicrobial drugs.

Category	Group of drugs	Name of the repositioned drug	Synergistic antimicrobial drug	Spectrum of activity	Ref.
Medicines used in oncology	Cytostatics	Mitoxantrone	Colistin	*P. aeruginosa*	Torres et al. (2018)
		Mitotane	Polymyxin B	*A. baumannii, P. aeruginosa, K. pneumoniae*	Tran et al. (2018), Gontijo et al. (2022)
	Selective estrogen receptor modulators	Toremifene	Polymyxin B	*P. aeruginosa*	Hussein et al. (2017)
Medicines used in gastroenterology	Proton pump inhibitors	Omeprazole	Erythromycin	*S. aureus*	Mahfouz et al. (2023)
	Synthetic piperidine derivative	Loperamide	Minocycline	*Salmonella* sp.	Brown (2015)
		Loperamide	Novobiocin	*E. coli*	Brown (2015)
Medicines used in therapy against pain	NSAIDs	Acetylsalicylic acid	Vancomycin, cefuroxime, chloramphenicol	*S. aureus* (include MRSA)	Brown (2015); Chan et al. (2017), Barbarossa et al. (2022)
		Acetylsalicylic acid	Linezolid	*Staphylococcus* sp.	Maarouf et al. (2021)
		Ibuprofen	Cefuroxime, chloramphenicol	*S. aureus* (include MRSA)	Chan et al. (2017)
		Ibuprofen	Linezolid	*Staphylococcus* sp.	Maarouf et al. (2021)
		Ibuprofen	Ceftazidime	*P. aeruginosa*	Chen et al. (2023)
		Diclofenac	Streptomycin	*E. coli, Mycobacterium* spp.	Foletto et al. (2021a)
		Diclofenac	Gentamicin	*Listeria* spp.	Brown (2015)
			Oxacillin	*S. aureus* (include MRSA)	Queiroz et al. (2021)
		Celecoxib	Polymyxin B	*S. aureus* (include MRSA)	Krishnamurthy et al. (2019)
Medicines used to treat cardiovascular disorders	Calcium channel blockers	Amlodipine	Tetracycline	*A. baumannii*	Ugurel and Turgut-Balik (2023)
		Amlodipine	Imipenem	*A. baumannii*	Hu et al. (2018)
		Verapamil	Amikacin, tigecycline, cefoxitin	*M. abscessus*	Mudde et al. (2022)
	β-blockers	Carvedilol	Gentamicin	*A. baumannii*	Ugurel and Turgut-Balik (2023)
		Carvedilol	Ciprofloxacin	*S. aureus*	Zawadzka et al. (2019)
Medicines used in neurological therapy and psychiatry	Antidepressants	Sertraline	Fluoroquinolones, aminoglycosides	*S. aureus*	Barbarossa et al. (2022)
		Sertraline	Gentamicin, erythromycin	*P. aeruginosa, E. coli*	Barbarossa et al. (2022)
		Sertraline	Amoxicillin, clarithromycin, tetracycline, metronidazole	*H. pylori*	Barbarossa et al. (2022)
		Sertraline	Polymyxin B	*A. baumannii, E. coli, K. pneumoniae, Enterococcus* spp.	Otto et al. (2019), Foletto et al. (2021a)
		Fluoxetine, Paroxetine	Ciprofloxacin	*E. coli, A. baumannii, K. pneumoniae, E. faecium*	Foletto et al. (2020), Foletto et al. (2021b)
		Amitriptyline	Colistin	*K. pneumoniae*	Barbarossa et al. (2022), Ugurel and Turgut-Balik (2023)
Medicines used in epilepsy		Valproic acid	Isoniazid, rifampicin	*Mycobacterium* spp.	Rodrigues et al. (2020)
Medicines used in neurodegenerative diseases		Memantine	Ampicillin	*E. coli*	Yu et al. (2015)
Medicines used in parasitic infections	Salicylanilide derivatives	Niclosamide	Colistin	*A. baumannii, K. pneumoniae*	Barbarossa et al. (2022)
		Oxyclozanide, rafoxanide, closantel	Colistin	*P. aeruginosa, A. baumannii, K. pneumoniae, E. coli, E. cloacae*	Tran et al. (2016), Domalaon et al. (2019)
Medicines used in metabolic disorders	Antidiabetic drugs	Acarbose	Isoniazid, ethambutol	*M. tuberculosis*	Sharma et al. (2023)
	Statins	Simvastatin	Rifampicin, isoniazid, pyrazinamide	*M. tuberculosis*	Skerry et al. (2014)
Other drugs		Ebselen	Mupirocin, fusidic acid, retapamulin, daptomycin	*Staphylococcus* spp.	Maslanka and Mucha (2023)
		Ebselen	Isoniazid	*M. tuberculosis*	Padiadpu et al. (2016), Sharma et al. (2023)
		Auranofin	Azithromycin, ceftriaxone, cefixime, tetracycline	*N. gonorrhoeae*	Elkashif and Seleem (2020)
		Auranofin	Colistin	*P. aeruginosa*	Torres et al. (2018)
		Auranofin	Colistin and ceftazidime, colistin and rifabutin	*K. pneumoniae, A. baumannii, P. aeruginosa, C. freundii, E. cloacae, E. coli*	Sun et al. (2016)
		Cyclosporine A	Ethambutol, isoniazid	*M. tuberculosis*	Kumar et al. (2019b)

However, research is needed to ensure that the transferred drug reaches its expected site of action and to limit drug-related side effects while maintaining its antimicrobial activity. An additional problem is also possible drug interactions that could occur between drugs showing synergism and drugs of patients with concomitant diseases. In the case of some compounds, such as acarbose or some antidepressants, the impact of the repositioned drug on the patient’s microflora is also important. As a result of combining different classes of drugs in therapy, the increase in antibiotic resistance may also accelerate (Tarín-Pelló et al., 2023).

Many of the presented drugs exhibit antibacterial activity at concentrations higher than those available in human serum. However, many of them may affect the activity of the human immune system, which would indicate the possibility of using such drugs as adjuvants in the treatment of infections. Among the drugs described, those used in metabolic disorders (e.g., seem to have the greatest repositioning potential due to their well-known pleiotropic effects, pharmacological profile and side effects) in fact, preclinical and clinical studies seem to indicate that this group of drugs has the greatest potential for repositioning in new indications. Selective serotonin reuptake inhibitors also show great promise in repositioning themselves in the treatment of infectious diseases due to their diverse mechanisms of antimicrobial action.

Although the presented examples of drug candidates for repositioning and their activity profile raise great hopes, it should be emphasized that most of the studies are *in vitro* studies, which do not allow drawing far-reaching conclusions. Moreover, more detailed research is needed, including *in vivo* animal studies and clinical trials. They will answer questions about the precise pharmacokinetics, pharmacodynamics, effectiveness, stability and safety of the new drug. The effectiveness of the new, repositioned drug is also related to the selection of its appropriate dose and form of the drug, taking into account the possible side effects it may cause. In the era of increasing antibiotic resistance, it is also reasonable to determine whether a new compound may cause resistance to itself or increase resistance to antibiotics used in therapy. Ongoing screening using patient isolates and drug candidates, also taking into account possible synergism with existing antibiotics, may identify treatments for severe infections. Additionally, it will reduce the time, costs and risks associated with the introduction of new antibacterial substances. It also brings hope given the scarcity of effective therapeutic options for treating infectious diseases.

## 4 Conclusion

The process of drug repurposing can be a rapid and effective method for discovering new antibacterial substances, as well as a solution to the problem of increasing antibiotic resistance, especially in the face of the small number of drug discoveries. This procedure is faster compared to *de novo* development of new antimicrobials. The review presents current data on the potential for repositioning drugs from various therapeutic groups in infectious diseases and summarizes the chance of their synergistic use in combination with antibiotics. The spectrum of their action and the possibility of use in the case of infections caused by multidrug-resistant bacteria were also taken into account.

Much research is still needed to identify potential repositioning candidates in infectious bacterial diseases in preclinical and clinical studies. Nevertheless, this research is necessary and antibacterial therapy in the treatment of infections, often caused by multidrug-resistant strains of bacteria, using repositioned drugs is promising and has great commercial potential.
